# Cluster Analysis Reveals Subgroups with Different Risk Profiles and Sickness Absence Patterns in an Occupational Health Cohort

**DOI:** 10.1007/s10926-025-10319-x

**Published:** 2025-07-29

**Authors:** Anniina Anttila, Mikko Nuutinen, Riikka-Leena Leskelä, Mark van Gils, Anu Pekki, Riitta Sauni

**Affiliations:** 1 Tampere University, Arvo Ylpön Katu 34Tampereen Yliopisto, PL 100, 33014 Tampere, Finland; 2 Finla Työterveys, PL 42, 33211 Tampere, Finland; 3 Nordic Healthcare Group, Keilaniementie 1, 02150 Espoo, Finland

**Keywords:** Work disability, Screening, Occupational health, Unsupervised learning, Employee characteristics

## Abstract

**Purpose:**

Using unsupervised and supervised machine learning methods, we aimed to identify clinically relevant groups of employees with similar characteristics and analyze the association of long and short sickness absence periods with these groups.

**Methods:**

The participants were 12,099 employees of various occupations in Finnish companies. The data comprised 104 variables from medical records including data on sickness absences and a questionnaire used between 2011 and 2019 in health examinations. The latent dimensions for the employees were defined by principal component analysis to reduce the number of variables. Clusters were calculated using the K-means algorithm from datapoints expressed by the resulting five principal components. Logistic regression analyses were used to assess the associations of the clusters with long (> 30 days) and repetitive short (1–10 days) sickness absence (SA) episodes.

**Results:**

Employees in cluster one indicated positive managerial performance and workplace atmosphere, and employees had the least of both short and long SA. Cluster two indicated deficiencies related to managerial performance and workplace atmosphere. Cluster three had deficiencies mainly related to mood and depression and cluster four had cardiovascular diseases. Employees in cluster five reported many symptoms, especially dizziness and sensory symptoms, and had the highest occurrence of repetitive short SA. Cluster six indicated deficiencies related to work ability and had the highest occurrence of a long SA episode during follow-up.

**Conclusion:**

Unsupervised and supervised machine learning methods identified six clinically coherent employee clusters, providing information on typical combinations of characteristics and risk profiles of sickness absence.

**Supplementary Information:**

The online version contains supplementary material available at 10.1007/s10926-025-10319-x.

## Introduction

Work disability causes considerable financial challenges for society, especially in countries with a strong welfare system. Besides the public expenditure on disability benefits, diseases causing work disability result in increased health care costs with a simultaneous loss of tax income from working-aged adults unable to work. The financial impact of work disability on workplaces through loss of productivity and disruptions of workflow are also considerable. The importance of taking all possible measures to tackle the problem of work disability has increased as the workforce ages and is widely recognized [1].

Common mental and behavioral disorders have become the most important diagnosis group of work disability in Finland in recent years, causing 35.7% of reimbursed sickness absence (SA) days in 2022. Musculoskeletal diseases, which were the most common cause of SAs up to 2020, caused 25.2% of reimbursed SA days in 2022 [2].

The SA risk has been shown to increase in accordance with certain lifestyle factors such as low physical activity, smoking, and heavy episodic drinking [3]. Simultaneous exposure to multiple lifestyle risk factors has been shown to further increase risk of sick leave and reduce work ability [4, 5]. Psychosocial work factors such as low job control and low worktime control, in addition to several somatic reasons, have also shown association with increased SAs [6].

The coexistence of different work disability risk factors is not uncommon in the same individual. Data analysis methods, such as clustering, can identify individuals with similar exposure and work disability characteristics. Clustering methods may reveal possible structures in the data in a data-driven way without pre-established hypotheses [7]. For example, multivariate modeling, commonly used in many analyses, is based on a pre-defined hypothesis about the relationship between independent variables and the dependent variable, and the model tests the significance of this relationship. In contrast, cluster analysis allows for new discoveries and visualization of previously unknown structures, especially when the data are sufficiently large and comprehensive.

Latent class analysis was used in a study using data from Finland, France and the United Kingdom to identify subgroups of diabetic employees with different work disability outcomes. The study found approximately half of the diabetic employees belonged to a subgroup with a clustering of simultaneous and a high prevalence chronic somatic diseases, psychological symptoms, obesity, physical inactivity and, surprisingly, alcohol abstinence and an associated high risk of work disability, while the other half belonged to a subgroup with a more favorable risk profile [8].

Cluster analysis was utilized in a recent Danish study to examine joint associations between different combinations of psychosocial risk factors with long-term SAs as the outcome [9]. They found that a poor score on several psychosocial work factors increased the risk of a long-term SA, while the potential adverse effects of scoring poor on one or two factors could be outweighed by favorable scores on several other psychosocial factors.

The use of data clustering methods is still limited in studies on occupational health and work disability. Our first goal in this study was to identify groups of similar employees from a heterogeneous group based on characteristics and risk factors using unsupervised machine learning methods on extensive survey data. Using unsupervised methods, we discovered employee groups from data without pre-defined labels or correct answers. In addition, we applied supervised machine learning methods and cluster membership information for exploring the characteristics of the clusters in more detail. Second, we analyzed how the groups are associated with long and short SA periods. We hypothesized that a data-driven approach would lead to distinct and clinically relevant employee profiles that are associated with long or short SA periods or both. Previously unidentified employee profiles, that depict typical combinations of risk factors and characteristics, could provide valuable information for occupational health professionals in planning appropriate personalized interventions targeted at the specific properties of each cluster. This could help to focus the limited resources of occupational health more efficiently.

## Methods

### Study Design and Settings

We had access to the electronic medical records of one large occupational healthcare provider (Finla). The study design was a retrospective analysis of prospectively collected data on SAs, diagnoses and contacts with occupational health professionals and questionnaires used in periodic health examinations. The health questionnaire included a wide variety of questions regarding health habits, working conditions, previous illnesses and symptoms, and questionnaires for screening depression and cardiovascular risk, among others.

The research plan was approved by the Finnish data authority Findata (THL/1850/14.02.00/2022). Ethics approval is not required in Finland for retrospective registry studies with pseudonymized data such as ours.

### Study Population

The study’s participants were employees of various companies who were entitled to occupational health services provided by Finla between 2009 and 2021 and who completed the screening questionnaire between 2011 and 2019. Even though many of the participants also visited occupational health as patients, screening was targeted at employees regardless of their current health. Therefore, we shall use the term employee throughout this study.

We included the first response of employees who had completed the questionnaire more than once from the original 22,919 questionnaires. We excluded questionnaires that could not be matched to the service use register, lacked a registered response time or had been completed later than 2 years before the beginning of COVID-19 restrictions, because the pandemic would have influenced SAs during the follow-up time. We landed at the final sample size of 12,099 employees after these adjustments. The online resource material (Figure S1) presents the study flow.

### Statistical Methods

This study’s main aim was to identify different subgroups of employees based on factors of demography, job description, health habits, diseases and symptoms, depressive symptoms, psychosocial factors, work ability, and supervisor performance and workplace atmosphere. In the first step, we defined the latent dimensions for the employee characteristics by principal component analysis (PCA) [10], whose purpose was to reduce the number of variables. In the second step, we calculated the clusters from datapoints expressed by the latent dimensions using the K-means algorithm [11]. The PCA was applied for polychoric correlation matrices instead of Pearson linear correlations. Pearson linear correlation assumes continuous and normal variables. The questionnaire data are mainly binary and ordinal category variables. Polychoric correlations can be calculated without the assumption of normal data [12].

The PCA was performed using all variables (Online Resource Table S1 and the chapter “Collected variables”). The advantage of using the PCA components as input to the clustering algorithm instead of the original variables is that the components are continuous and orthogonal to each other as well as centered around zero with similar standard deviations. Furthermore, the high dimensionality of the original data is reduced to a few latent dimensions (five in our study, please see the “Results” section). As standard PCA, the K-means clustering algorithm may benefit if the data are continuous and variables are on comparable scales to avoid bias towards features with large value ranges. High dimensionality could result in unstable solutions because a fixed number of data points becomes increasingly sparse as the dimensionality increases. Sampling adequacy of the variable set and each individual variable for the PCA analysis was measured by the Kaiser–Meyer–Olkin (KMO) measure [13]. The second test of data suitability for the PCA was the Bartlett’s test of sphericity measuring adequate correlations between variables [14].

One challenge in the PCA and the cluster analyses is to choose the number of components and clusters for the final solution. In this study, Eigenvalues were used in the scree plot to search for the optimal number of principal components. We used the measures of average silhouette width [15] and adjusted the Rand Index (ARI) [16] to search for an optimal clustering solution. The average silhouette width measures compactness and separation of the clusters. The ARI is a stability measure for assessing whether clustering different datasets sampled from the same underlying joint distribution results in the same clusters. Average ARI values were obtained by running K-means clustering on 100 bootstrapped samples across 2 to 10 clusters. Eigenvalues correspond to the amount of variation explained by each component, that is, how much information each component contains. The analysis of the scree plot is a typical method used in previous studies [17, 18].

The resulting clusters were analyzed by three methods. (1) Descriptive statistics were used to compare the clusters’ characteristics. (2) Supervised classification models (XGBoost [19, 20]) and Shapley values [21, 22] were next used to identify the variables that contributed the most in the characterization of the different clusters. Each cluster had its own classification model that was trained to discriminate the employees of the cluster from all other employees. All variables included in the PCA (Online Resource Table S1) were used as predictors for the classification models. (3) Finally, logistic regression analyses were used to assess the associations of the clusters for long and short SA episodes. The outcome of short SA episodes was defined as more than five short (1–10 days) SA episodes during a two-year follow-up time. The outcome of long SA episodes was defined as one or more long (> 30 days) SA episodes during a 2-year follow-up time. The follow-up time began on the date of response to the questionnaire. The episodes were combined into one SA episode if there were 1–3 days between the end and start of two SA episodes. Online Resource Figure S2 presents the analytical pipeline and the inputs and outcomes of different steps.

All statistical analyses were done using Python (version 3.8.12) and R (4.0.5). K-means clustering and classifiers were done by sklearn library (1.0.2). Principal component analysis was done by psych library (2.3.9).

## Results

### Study Population

The study comprised 12,099 employees. Online Resource Table S1 presents the properties of the employees with or without long SA periods during follow-up. The average age was 42.7 years (SD 11.5; range 16–69). Of the respondents, 42.7% (N = 5163) were female. Altogether, 16.9% worked as a supervisor, and 43.2% worked in a blue-collar position. Furthermore, 23.7% of them performed shift work, and 6.1% performed night work. The most common self-reported diseases were insomnia (15.1%), musculoskeletal disease (13.0%) and hypertension (12.5%).

### Cluster Analysis

104 variables were used for the PCA (Online Resource Table S1). The KMO coefficient for the variable set was 0.86, and for each individual variable 0.64—0.98, indicating good sampling adequacy [23]. Bartlett’s test of sphericity was significant p < 0.001, indicating that the correlation matrix for the variable set was suitable for the PCA.

Figure S3 shows the eigenvalues for the 10 first principal components (scree plot). The ‘knee’ in the scree plot can be noticed after four or five components. We selected five components for further analyses. These five principal components explained 14.7%, 6.4%, 5.7%, 5.6% and 4.7% of the variation in the dataset.

Table 1 shows the top 10 variables with the largest loading values of the five principal components. Principal components can be interpreted as characterizations of different dimensions of the employees screened in occupational healthcare. For example, PC1 was excited by the variables such as “Felt that even help from my family and friends cannot ease my melancholy”, “Felt all joy has gone from life” and “Felt lonely”. That is, the variables of PC1 are related to mood and depression. PC2 was excited by the variables of “Management provides the necessary help and encouragement”, “Managerial work is fair and equal”, “There is a good spirit within our personnel” and “I have the possibility of self-reformation at my job”. PC3 was excited by the variables “Work ability has become worse”, “Does your disease hinder coping at work” and “Musculoskeletal disease”. PC4 was excited by the variable “Number of symptoms” and symptoms such as “Fainting, consciousness disturbances”, “Hearing impairment”, “Vision impairment”, “Dizziness” and “Arrhythmia”. PC5 was excited by the variables “Diabetes”, “Blood glucose elevated”, “Hypertension” and “Age”. We labeled the principal components according to the variables. The labels are mood and depression (PC1), Managerial performance and workplace atmosphere (PC2), Work ability (PC3), Dizziness and sensory symptoms (PC4), and Cardiovascular disease (PC5).

**Table 1:** The loadings of the variables sorted in descendant orders for five principal components (10 highest values for each principal component) in a Finnish occupational health cohort in 2011–2019

Item name	Mood and depression (PC1)	Managerial performance and workplace atmosphere (PC2)	Work ability (PC3)	Dizziness and sensory symptoms (PC4)	Cardiovascular disease (PC5)
Deps score	0.088				
Felt that even help from my family and friends cannot ease my melancholy	0.088				
Felt melancholic	0.088				
Felt all joy has gone from life	0.087				
During past 1 month often bothered by little interest or pleasure in doing things	0.085				
Felt impossible to find pleasure in life	0.083				
Felt lonely	0.081				
Felt hopeless about the future	0.076				
During past 1 month often bothered by feeling down, depressed or hopeless	0.073				
Joylessness, hopelessness symptom	0.073				
Management provides the necessary help and encouragement		0.154			
Managerial work is fair and equal		0.151			
My work is appreciated, and I am praised for a job well done		0.149			
There is a good spirit within our personnel		0.127			
I have the possibility of self-reformation at my job		0.125			
I can rely on the continuance of my employment		0.117			
I enjoy my job		0.115			
I have not witnessed bullying or harassment at our workplace		0.113			
My job is versatile		0.108			
I can influence the contents, pace, and hours in my job		0.095			
Work ability has become worse			0.181		
Does your symptom hinder coping at work			0.173		
Self-rated change in work ability			0.165		
Does your disease hinder coping at work			0.153		
Personal prognosis of work ability in current job 2 years from now			0.140		
Self-rated work ability			0.136		
Musculoskeletal disease			0.130		
Work ability has not changed			− 0.121		
Musculoskeletal symptom			0.115		
Pain symptom			0.095		
Fainting, consciousness disturbances symptom				0.226	
Number of symptoms				0.150	
Hearing impairment symptom				0.144	
Vision impairment symptom				0.139	
Dizziness symptom				0.138	
Memory problems symptom				0.129	
Arrhythmia symptom				0.126	
Learning difficulties symptom				0.124	
Weakness or clumsiness of limbs symptom				0.100	
Headache symptom				0.094	
Diabetes					0.193
Blood glucose elevated					0.189
Hypertension					0.176
Age					0.125
Blood pressure elevated					0.115
Cardiovascular disease					0.099
BMI					0.099
Cholesterol elevated					0.091
Number of diseases					0.079
Ear disease, hearing loss					0.079

**Fig. 1:**
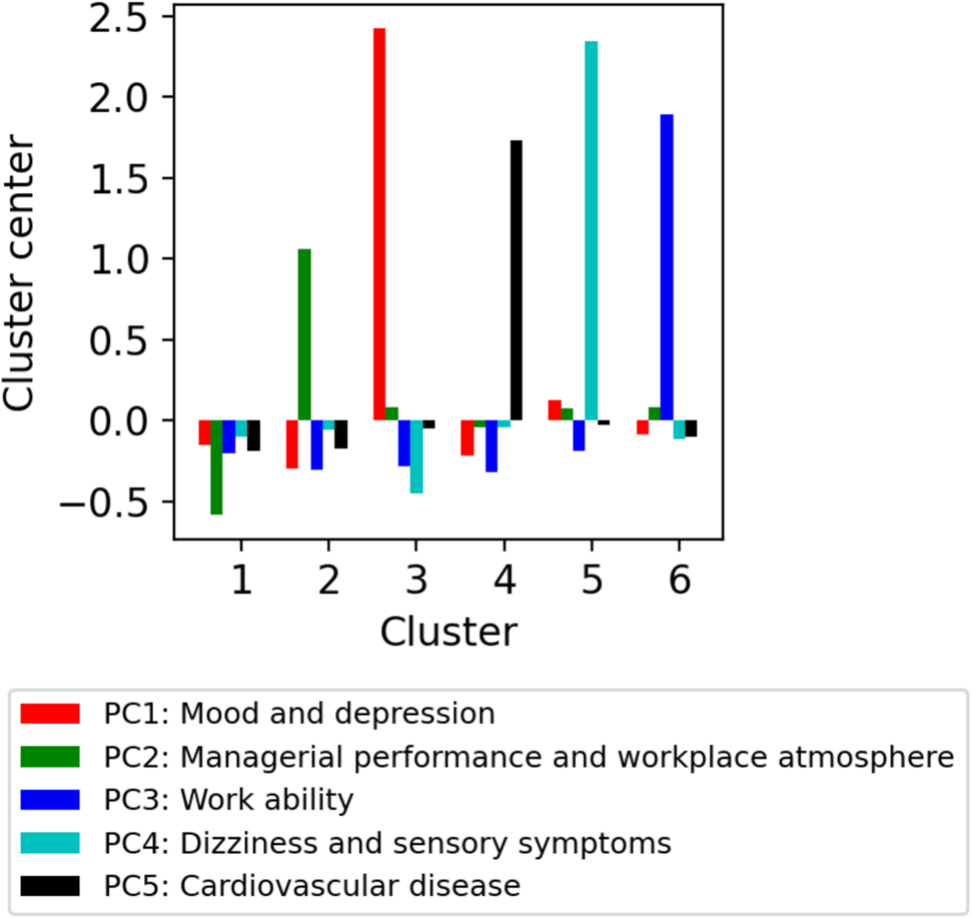
Cluster centers in the space of the 5 principal components in a Finnish occupational health cohort from 2011 to 2019

The data points presented in the space of the five principal components were clustered by the K-means algorithm. Figure 1 shows the center values of the principal components for the six clusters. The number of clusters was selected based on the Silhouette and ARI scores (Figures S4 and S5). The numbers of employees in clusters one, two, three, four, five and six were 5503, 2863, 800, 989, 543 and 1401, respectively. Table 2 shows descriptive statistics of background variables and their distribution in different clusters; statistics of a more extensive set of variables are given in Online Resource table S2. Figure 1 shows that employees located in cluster one indicated positive managerial performance and workplace atmosphere, healthy employees (PC2). Cluster two indicates deficiencies related to managerial performance and workplace atmosphere (PC2). Employees in cluster three have deficiencies related to mood and depression (PC1). Cluster four indicates cardiovascular diseases (PC5). Cluster five indicates experiencing symptoms such as dizziness and sensory symptoms (PC4). Cluster six indicates experiences of diminished work ability (PC3).

**Table 2:** Descriptive statistics of background variables and their distribution in the different clusters in a Finnish occupational health cohort from 2011 to 2019

	Item name	All	Cluster 1 Healthy employees	Cluster 2 Managerial performance and workplace atmosphere	Cluster 3 Mood and depression	Cluster 4 Cardiovascular diseases	Cluster 5 Dizziness and sensory symptoms	Cluster 6 Work ability
	Employees, n	12,099	5503	2863	800	989	543	1401
Demography	Gender, n (%)	5163 (42.67)	2265 (41.16)	1244 (43.45)	390 (48.75)	293 (29.63)	295 (54.33)	676 (48.25)
	Age, mean (std)	42.69 (11.45)	40.78 (11.33)	40.85 (10.64)	42.55 (11.01)	53.05 (7.95)	43.42 (11.25)	46.4 (11.12)
	BMI, mean (std)	26.13 (4.52)	25.51 (4.15)	26.01 (4.34)	26.64 (5.07)	29.38 (5.28)	25.84 (4.57)	26.33 (4.33)
	Smoker, n (%)	3261 (26.95)	1450 (26.35)	733 (25.6)	277 (34.62)	217 (21.94)	159 (29.28)	425 (30.34)
Job description	Supervisor, n (%)	2046 (16.91)	1219 (22.15)	222 (7.75)	113 (14.12)	190 (19.21)	51 (9.39)	251 (17.92)
	White-collar worker, n (%)	5231 (43.23)	2731 (49.63)	1003 (35.03)	336 (42.0)	457 (46.21)	231 (42.54)	473 (33.76)
	Blue-collar worker, n (%)	6484 (53.59)	2605 (47.34)	1784 (62.31)	439 (54.87)	487 (49.24)	296 (54.51)	873 (62.31)
	Shift work, n (%)	2871 (23.73)	1201 (21.82)	777 (27.14)	204 (25.5)	191 (19.31)	122 (22.47)	376 (26.84)
	Night work, n (%)	740 (6.12)	238 (4.32)	293 (10.23)	50 (6.25)	42 (4.25)	43 (7.92)	74 (5.28)
	Part-time work, n (%)	1603 (13.25)	764 (13.88)	289 (10.09)	138 (17.25)	82 (8.29)	82 (15.1)	248 (17.7)
Diseases	Number of diseases, mean (std)	1.47 (1.6)	0.95 (1.16)	1.1 (1.26)	2.39 (1.89)	2.97 (1.94)	2.53 (1.96)	2.26 (1.68)
	Number of symptoms, mean (std)	2.87 (2.78)	1.85 (1.73)	2.38 (1.99)	5.68 (3.3)	2.33 (2.21)	8.79 (3.69)	4.3 (2.62)
	Asthma, pulmonary disease, n (%)	814 (6.73)	326 (5.92)	164 (5.73)	65 (8.12)	90 (9.1)	63 (11.6)	106 (7.57)
	Diabetes, n (%)	419 (3.46)	9 (0.16)	12 (0.42)	21 (2.62)	348 (35.19)	7 (1.29)	22 (1.57)
	Common mental disease, n (%)	757 (6.26)	172 (3.13)	88 (3.07)	269 (33.62)	59 (5.97)	70 (12.89)	99 (7.07)
	Cardiovascular disease, n (%)	624 (5.16)	104 (1.89)	99 (3.46)	51 (6.38)	208 (21.03)	84 (15.47)	78 (5.57)
	Cancer, n (%)	244 (2.02)	58 (1.05)	51 (1.78)	16 (2.0)	65 (6.57)	12 (2.21)	42 (3.0)
	Musculoskeletal disease, n (%)	1574 (13.01)	374 (6.8)	239 (8.35)	113 (14.12)	159 (16.08)	95 (17.5)	594 (42.4)
	Insomnia, tiredness, n (%)	1828 (15.11)	435 (7.9)	333 (11.63)	359 (44.88)	117 (11.83)	183 (33.7)	401 (28.62)
	Hypertension, n (%)	1508 (12.46)	291 (5.29)	189 (6.6)	121 (15.12)	614 (62.08)	59 (10.87)	234 (16.7)
Sickness absences	SA repetitive short periods (next 2 years), n (%)	1055 (8.72)	366 (6.65)	296 (10.34)	84 (10.5)	70 (7.08)	75 (13.81)	164 (11.71)
	SA long period (next 2 years), n (%)	716 (5.92)	199 (3.62)	137 (4.79)	75 (9.38)	79 (7.99)	44 (8.1)	182 (12.99)

Repetitive short periods were defined as more than five short (1–10 days) sickness absence episodes and long periods as over 30 consecutive sickness absence days during a 2-year follow-up time

Figures S6–S11 present Shapley values for the 15 most important variables predicting cluster membership. Shapley values were calculated from the classification models that were trained for discriminating each cluster from all other clusters. 104 variables were used for training the classifiers (Online Resource Table S1). It should be noted that with Cluster one (healthy employees), the high values (red points) of the listed variables decrease the probability of cluster membership.

Age was among the most important variables defining clusters three and four, where younger age increased the likelihood of membership in cluster three and older age in cluster four. The most important variable for defining membership in cluster six was reporting deteriorating work ability, whereas the experience of deteriorating work ability was among the most important variables diminishing the probability of cluster membership in clusters one and two. A high score in the depression screening questionnaire (DEPS) was the most important variable defining membership of cluster three, whereas a high DEPS score diminished the probability of membership in clusters one, two, five and six. The DEPS score was among the 15 most important defining variables in all clusters except cluster four.

### Associations of Clusters with Long and Short Sickness Absence Periods

**Fig. 2:**
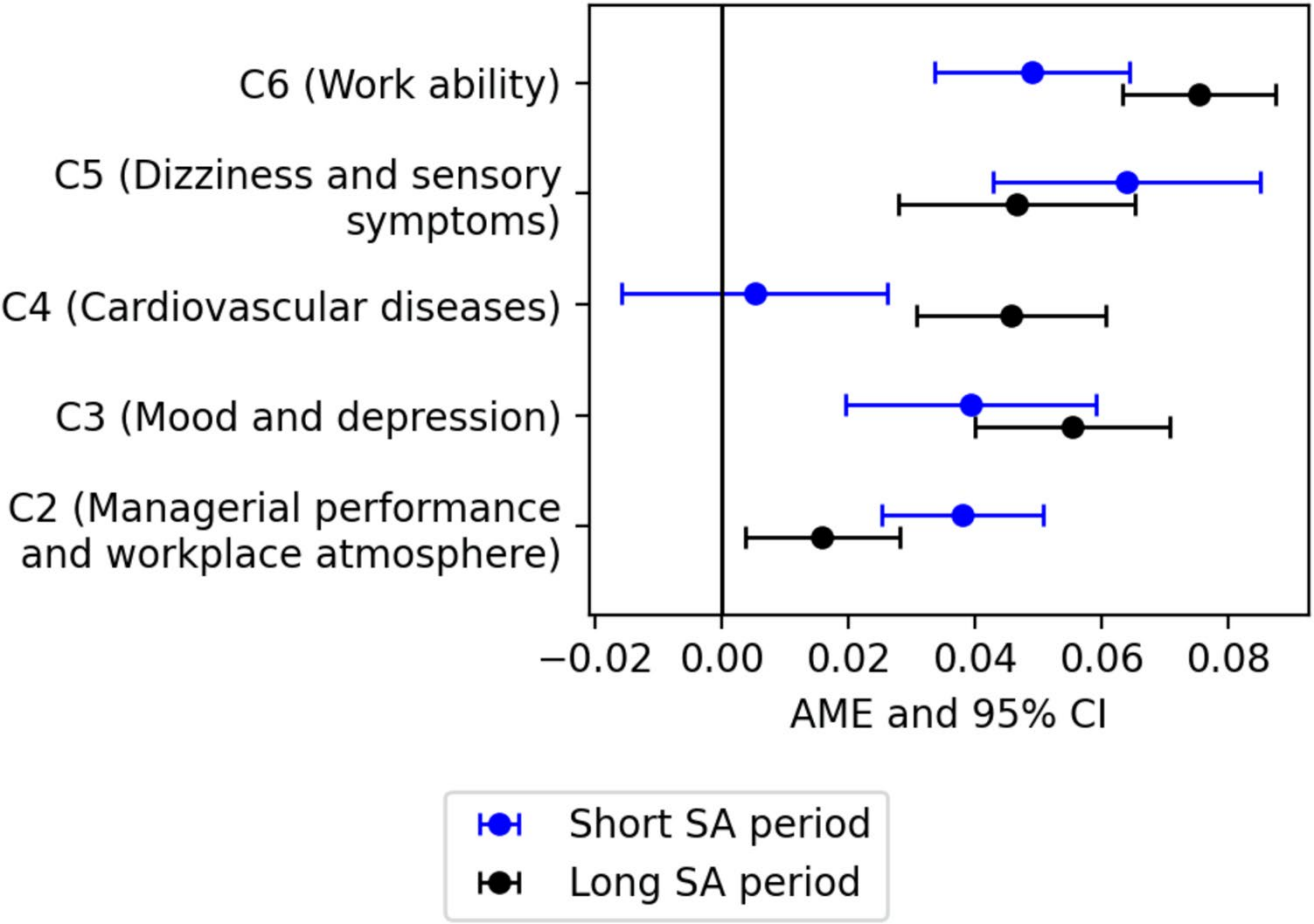
Associations of clusters with repetitive short and long sickness absences in a Finnish occupational health cohort in 2011–2019. Note. average marginal effects (AME) values and 95% confidence intervals of the logistic regression models of the outcomes more than five short (1–10 days) sickness absence episodes during a 2-year follow-up time or one or more long (> 30 days) sickness absence episode during a 2-year follow-up time. The reference cluster was the healthy cluster (Cluster 3)

Figure 2 shows AME (95% CI) values for the clusters for the outcomes of long and repetitive short SA episodes in the forthcoming 2-year period. Figure S12 presents OR (95% CI) values. The reference cluster was the healthy cluster (Cluster one), with 6.7% of the cluster members having more than five short SA episodes and 3.6% having at least one long SA episode during a 2-year follow-up (Table 2). The strongest association for long SA episodes was for Cluster six, 13.0% of whose members had at least one long SA episode during a 2-year follow-up. The membership of this cluster was defined by the experience of their work ability becoming worse and reporting that their symptoms and diseases hindered coping at work (Figure S11). They typically experienced musculoskeletal symptoms and pain, yet their score on a depression screening questionnaire seldom portrayed increased depressive symptoms. The AME (95% CI) was 0.08 (0.06–0.09), meaning that the probability of a long SA period increased by 8 percentage points when Cluster six is compared to Cluster one (Healthy employees).

In clusters two and five, the increase in the probability of short SA episodes was greater than the increase in the probability of long SA episodes (AME values: 3.8% vs. 1.6% and 6.4% vs. 4.7%) with 10.3% of cluster two and 13.8% of cluster five members having more than five short SA episodes in a 2-year follow-up. The most important variables defining membership in cluster five were reporting many symptoms, especially dizziness or fainting, but not necessarily having any diagnosed diseases. A large AUDIT score, which indicates excess alcohol use, also increased the probability of membership in this group. Cluster two comprised employees who generally reported a negative experience in questions on managerial performance and workplace atmosphere.

For members of cluster four, among whom cardiovascular diseases, hypertension and diabetes were the most common variables defining cluster membership, the probability was increased only in long SA episodes.

## Discussion

In this study, we analyzed data from questionnaires used in health surveillance combined with data on occupational healthcare visits from electronic medical records. We used unsupervised methods (principal component analysis and K-means clustering) to identify groups of individuals (clusters) with similar characteristics and risk factors. The clusters were characterized by supervised machine learning and explainable artificial intelligence methods (XGboost classifier and Shapley values). The results were six clinically relevant clusters with diverse employee profiles.

In addition to defining the employee clusters, we found that these clusters differed in the incidence of repetitive short and long SAs. This is a central revelation of our study: the clusters portray different combinations of defining variables, but the recognition of these employee profiles can also help tackle the distinct SA profile associated with each group.

Thirteen of the 15 most important variables defining cluster membership in our reference cluster (cluster one) were positive answers regarding managerial performance and workplace atmosphere, the most important being the experience that their work was appreciated. This cluster had the smallest number of both short and long SAs. Members of cluster two, however, generally gave a negative answer to questions on themes of managerial performance and workplace atmosphere. Members of this cluster typically did not have an elevated depression screening questionnaire (DEPS) score or report deteriorating work ability but in this group, the incidence of short SAs was significantly higher than in the reference cluster.

Previous studies have shown leadership quality to be associated with the incidence of SAs [24, 25]. This effect is probably partly due not only to atmosphere and communication issues but also to the superior’s willingness to facilitate job accommodations in situations of diminished work ability, which should also be supported by organizational disability management policies [26–28].

Members of cluster six typically reported that their work ability had deteriorated and that their symptoms and diseases hindered coping at work. They also typically experienced musculoskeletal symptoms and pain, with an over sixfold incidence of musculoskeletal disease compared to the reference cluster. This cluster showed both the highest incidence of long SA periods and the second highest incidence of short SA episodes in the follow-up time. Our findings are in line with previous studies that support the value of self-reported work ability in predicting health-related exit and absence from work [29]. Musculoskeletal symptoms and pain have also been shown to be important predictors of SA [30, 31].

Members of cluster four typically had cardiovascular diseases and risk factors such as elevated blood pressure, blood sugar or cholesterol levels. They typically reported that their symptoms did not hinder coping at work. Nevertheless, the probability of long SAs in a 2-year follow-up was significantly increased in this cluster. This could indicate that the onset of work disability tends to be quite abrupt among employees with cardiovascular risk factors—that is, the work disability risk is not subjectively recognized. Dietary habits and exercise should not be forgotten in the quest for improving work ability, because they play a role not only in the prevention of cardiovascular disease and diabetes but also in obesity, a recognized work disability risk [3, 32, 33]. Exercise has been shown to decrease work disability in overweight individuals [34], and moderate exercise decreased SA spells overall and for both musculoskeletal and mental causes separately in a study of Finnish municipal employees [35]. Some evidence exists that workplace health promotion programs can have a positive effect on work-related outcomes such as absenteeism [34, 36, 37].

Despite the intriguing combinations of characteristics that we found to coexist in a large dataset and the employee profiles they portray, it must be noted that the clustering methods simply identify variables that increase or decrease the likelihood of membership in a given cluster. There is always substantial variation between individuals within clusters and despite the demonstrated associations, causality between cluster membership and sickness absence profiles cannot be presumed.

One of this study’s strengths was having access to both long and short SA data beginning from the first day of work disability. These data are seldom available in studies on employees of various companies and occupations. Another strength was the variety of themes within the questionnaire that could be linked to data on service use and SAs. This made it possible to use the full capacity of modern data analysis methods to recognize associations we would not have been able to find with more traditional methods or a more restricted data source.

We collected our data from a single Finnish occupational health service provider, which must be considered a limitation of this study. However, the distribution of industries among employees entitled to services at Finla Occupational Health is generally similar to that of Finnish companies (Figure S13). This improves the generalizability of the results, especially in a Finnish setting. Clustering methods have not been widely used in occupational health previously, so follow-up research is needed. A similar study setting in a different population would provide valuable added information and possibly validation to our findings.

The results of our study imply that utilizing clustering methods can pave the way towards effective interventions aimed at the characteristics of employees in a given population. Clustering methods could be used in constructing a company-level report to help occupational health professionals communicate the SA risks and needs of preventive measures in the future. A study on interventions targeting the needs of employee clusters would be an interesting focus of further research and could potentially help implement our results in the everyday work of occupational health personnel to prevent work disability.

## Conclusions

With the aging of the workforce and the constant need to maintain work ability, cluster analysis is a powerful tool for analyzing the accumulating data in occupational health. The resulting insight into risk factors and profiles of work disability could help to initiate further development of interventions best suited to the needs of each subgroup, enabling a more efficient focus of the limited resources in occupational health. It should be acknowledged that according to the results in this study population, employees with cardiovascular and metabolic risk factors might not recognize their increased work disability risk. For them, the most important interventions to avoid work disability are promoting a healthy lifestyle and an efficient and active treatment of somatic illnesses. Furthermore, despite the increased risk of SAs for some employee groups, improving managerial performance and workplace atmosphere issues could be most impactful, instead of or in addition to health-related interventions.

## Supplementary Information

Below is the link to the electronic supplementary material.

Supplementary file 1.

## Data Availability

No additional data are available due to data privacy reasons.

## Declarations

### Protection of Research Participants

This study used solely secondary data retrieved from registers; individual participants could not be recognized.
