# Supplementary material for: Cluster Analysis Reveals Subgroups with Different Risk Profiles and Sickness Absence Patterns in an Occupational Health Cohort
Source: J Occup Rehabil. 2025 Jul 29;36(3):852–62. doi: 10.1007/s10926-025-10319-x (PMC13364802; doi:10.1007/s10926-025-10319-x)
Supplement: Supplementary file 1 [file 10926_2025_10319_MOESM1_ESM.docx]

Online Resource:

Cluster analysis reveals subgroups with different risk profiles and sickness absence patterns in an occupational health cohort

Journal of Occupational Rehabilitation

Anniina Anttila, Mikko Nuutinen, Riikka-Leena Leskelä, Mark van Gils, Anu Pekki, Riitta Sauni

corresponding author: Anniina Anttila, Tampere University and Finla Occupational Health, anniina.anttila@finla.fi

Table of Contents

[Collected variables 2](#_Toc200356530)

[Table S1. Variables and prevalence of long sickness absence period (> 30 days period forthcoming 24 months) and missing value rates. 5](#_Toc200356531)

[Table S2. Descriptive statistics of background variables and their distribution in the different clusters. 8](#_Toc200356532)

[Figure S1. Study flow. 11](#_Toc200356533)

[Figure S2. Analytical pipeline. 12](#_Toc200356534)

[Figure S3. Eigenvalues for the 10 first principal components of the data set. 13](#_Toc200356535)

[Figure S4. Silhouette score for the clustering solutions from 2 to 10 clusters 13](#_Toc200356536)

[Figure S5. Adjusted Rand Index (ARI) for the clustering solutions from 2 to 10 clusters. 14](#_Toc200356537)

[Figure S6. Shapley values (SHAP) for the 15 most important variables predicting cluster membership of Cluster 1 (Healthy employees). 15](#_Toc200356538)

[Figure S7. Shapley values (SHAP) for the 15 most important variables predicting cluster membership of Cluster 2 (Managerial performance and workplace atmosphere). 16](#_Toc200356539)

[Figure S8. Shapley values (SHAP) for the 15 most important variables predicting cluster membership of Cluster 3 (Mood and depression). 17](#_Toc200356540)

[Figure S9. Shapley values (SHAP) for the 15 most important variables predicting cluster membership of Cluster 4 (Cardiovascular diseases). 18](#_Toc200356541)

[Figure S10. Shapley values (SHAP) for the 15 most important variables predicting cluster membership of Cluster 5 (Dizziness and sensory symptoms). 19](#_Toc200356542)

[Figure S11. Shapley values (SHAP) for the 15 most important variables predicting cluster membership of Cluster 6 (Work ability). 20](#_Toc200356543)

[Figure S12. Associations of clusters with repetitive short and long sickness absences in a Finnish occupational health cohort in 2011–2019. 21](#_Toc200356544)

[Figure S13. The distribution of industries among employees using Finla Occupational Health services in 2019 and the Finnish working population in 2022. 22](#_Toc200356545)

## Collected variables

The variables were collected from the health questionnaires and the registries of occupational healthcare visits and sickness absences. At total of 104 variables were processed from the data sources.

Demography

The demographic variables were age and sex of employees.

Job description

The job description variables were collected from the questionnaires. The variables were supervisor position (yes/no), white-collar worker, blue-collar worker, shift work, night work, part-time work.

Measurements

The measurements that were collected from the questionnaires were BMI (body mass index), Elevated blood pressure (>130/80 mmHg, >140/85 mmHg in medical treatment), elevated cholesterol (total cholesterol >5.0 mmol/l and/or LDL cholesterol >3.0 mmol/l) and elevated blood sugar (fasting blood sugar 6.1 mmol/l or more, sugar stress test 7.8 mmol/l or more). The measurements that were collected from the registries of occupational healthcare visit data were systolic blood pressure, diastolic blood pressure and pulse.

Health habits

The health habits that were collected from the questionnaires were sleeping hours, smoking (current smoker yes/no and pack-years) and the AUDIT -questionnaire addressing alcohol use.

On eating habits, a sum variable (0-20p) was composed from answers to the following questions:

- Do your meals usually include the following: a) low-fat dairy or meat products (1p) b) high-fibre grain products (1p) c) fruit/vegetables/berries (1p)
- How often do you consume vegetables, fruit or berries: a) less than daily (1p), b)1-2 times daily (2p), c) several times a day (3p)
- When thirsty, I usually drink: a) soft drinks, juice or milk (1p), b) light soft drinks or light juice (2p), c) water (3p)
- I skip the main meal a) daily (1p), b) 1-2 times a week (2p), c) once a week/never (3p)
- How much do you believe your alcohol intake affects your weight control: a) substantially (1p), b) to some degree (2p), c) not at all (3p)
- I usually eat when: a) I have cravings (1p), b) I want to treat myself (2p), c) I feel stress, anxiety, loneliness or similar emotions or situations (3p), d) food is available or it is mealtime (4p), e) I am hungry (5p)

On exercise habits, we used answers to the question “how many times a week do you do the following exercise for at least half an hour” on a scale of not at all, once, twice, three or more times weekly: a) aerobic exercise, b) exercise in everyday situations (walking or cycling to work, gardening, vacuuming and other similar chores, etc), c) muscle exercise. In addition to the answers of aerobic, everyday exercise and muscle exercise separately, a sum variable (0-12p) was composed for exercise habits in all these fields so that each sub-area gets 0-3 points according to how many times a week an action was reported.

Diseases and symptoms

We used the answers to a self-reported checklist of 20 diseases from the questionnaire, in addition to which the added sum of reported diseases was used as an independent variable:

- Anemia
- Asthma/pulmonary disease
- Diabetes
- Epilepsy
- Skin disease
- Thyroid disorder
- Ear disease/hearing loss
- Common mental disease (depression, anxiety, panic attacks)
- Migraine
- Dyspepsia/gastric ulcer/irritable bowel
- Hemorrhoids/constipation
- Eye disease
- Varicose veins
- Cardiovascular disease
- Cancer
- Musculoskeletal disease
- Insomnia, tiredness
- Hypertension
- Other illness

We used the answers to a self-reported checklist of 19 symptoms in the questionnaire, in addition to which the added sum of reported symptoms was used as an independent variable:

- Eczema
- Cough, rhinitis
- Gastrointestinal symptoms
- Dizziness
- Fainting, consciousness disturbances
- Arrhythmia
- Anxiety or panic symptoms
- Gloominess, irritability, tearfulness
- Joylessness, hopelessness
- Problem of sex life
- Memory problems
- Concentration difficulties
- Learning difficulties
- Vision impairment
- Hearing impairment
- Weakness or clumsiness of limbs
- Headache
- Pain
- Musculoskeletal symptom

**Working conditions and mental health**

Depression questions

The 10 depression questions collected from the questionnaire were the questions of the DEPS questionnaire, which has been validated for screening depression and is widely used in Finland. The questions were “Suffered from insomnia”, “Felt melancholic”, “Felt everything was an effort”, “Felt unenergetic”, “Felt lonely”, “Felt hopeless about the future”, “Felt impossible to find pleasure in life”, “Had feelings of worthlessness”, “Felt all joy has gone from life” and “Felt that even help from my family and friends cannot ease my melancholy”. The response options were “Not at all”, “Some extent”, “Quite a lot” and “Very much”. For the models these were coded to the values of 0, 1, 2 and 3. In addition to answers to the separate questions, the full added score of DEPS was used as an independent variable. Also, as additional questions not included in DEPS, the questions “Do you feel squeezed empty of energy by your work” and “Do you feel stress (tension, restlessness, anxiety or sleeping difficulties caused by things constantly occupying your thoughts)” were included with the same answer options

Furthermore, data collected from the questionnaire were the two Whooley questions used in screening depression: “During the past month, have you often been bothered by feeling down, depressed or hopeless” and “During the past month, have you often been bothered by little interest or pleasure in doing things”, in which the response options were “No” or “Yes”. For the models these were coded to the values of 0 and 1.

Psychosocial questions

We used the answers to questions regarding the recipient’s current job on the scale “agree”, “partially agree”, “partially disagree” or “disagree”. The questions were “I enjoy my job”, “ The mental workload of my job is suitable for me”, “ I am familiar with the expectations and duties of my work tasks”, “My capabilities are proportional with my job’s demands”, “My work is appreciated, and I am praised for a job well done”, “My job is versatile”, “I can influence the contents, pace, and hours at my job”, and “I have the possibility of self-reformation at my job”.

Work ability questions

We used the answers to the following questions on work ability from the questionnaire:

- Do a) symptoms or b) diseases hinder coping at work
- Personal prognosis of work ability in 2 years in current job
- Do problems of sleep, alertness, stress or mood hinder coping at work
- Self-rated work ability (1-10)
- Self-rated change in work ability: has become worse/improved/no change
- The physical demands of my job are proportional with my capabilities agree/partially agree/partially disagree/disagree
- Self-rated recovery from work in general: good/varies/constantly worse/bad

Managerial performance and workplace atmosphere questions

We used the answers to the following questions regarding managerial performance and workplace atmosphere on the scale “agree”, “partially agree”, “partially disagree” and “disagree.

- The management provides me with help and encouragement when necessary
- The managerial work is fair and equal
- There is a good spirit within our personnel
- I have not witnessed bullying or harassment at my working community

## Table S1. Variables and prevalence of long sickness absence period (> 30 days period forthcoming 24 months) and missing value rates.

If more than 10% of samples were missing, the variable was excluded from the analysis. For the other variables, missing values were imputed by using k-Nearest Neighbour approach (1)

| **Item name** | **All** | **SA No** | **SA Yes** | **P-values** | **Missing values, n (%)** |
| --- | --- | --- | --- | --- | --- |
| Employees, n | 12099 | 11383 | 716 |  |  |
| Gender, n (%) | 5163 (42.67) | 4752 (41.75) | 411 (57.4) | <.001** | 0 (0.0%) |
| Age, mean (std) | 42.69 (11.45) | 42.44 (11.45) | 46.61 (10.77) | <.001** | 0 (0.0%) |
| Supervisor, n (%) | 2046 (16.91) | 1961 (17.23) | 85 (11.87) | <.001** | 0 (0.0%) |
| White-collar worker, n (%) | 5231 (43.23) | 5048 (44.35) | 183 (25.56) | <.001** | 0 (0.0%) |
| Blue-collar worker, n (%) | 6484 (53.59) | 5987 (52.6) | 497 (69.41) | <.001** | 0 (0.0%) |
| Shift work, n (%) | 2871 (23.73) | 2624 (23.05) | 247 (34.5) | <.001** | 0 (0.0%) |
| Night work, n (%) | 740 (6.12) | 683 (6.0) | 57 (7.96) | 0.037* | 0 (0.0%) |
| Part-time work, n (%) | 1603 (13.25) | 1479 (12.99) | 124 (17.32) | 0.001* | 0 (0.0%) |
| BMI, mean (std) | 26.52 (4.52) | 26.45 (4.47) | 27.65 (5.14) | <.001** | 67 (0.55%) |
| Blood pressure elevated, n (%) | 1332 (11.01) | 1243 (10.92) | 89 (12.43) | 0.218 | 0 (0.0%) |
| Cholesterol elevated, n (%) | 1961 (16.21) | 1827 (16.05) | 134 (18.72) | 0.067 | 0 (0.0%) |
| Blood glucose elevated, n (%) | 461 (3.81) | 414 (3.64) | 47 (6.56) | <.001** | 0 (0.0%) |
| Blood pressure dia, mean (std) | 84.76 (10.2) | 84.61 (10.2) | 86.49 (9.98) | <.001** | 7232 (59.77%) |
| Blood pressure sys, mean (std) | 133.71 (16.38) | 133.6 (16.39) | 135.07 (16.27) | 0.111 | 7215 (59.63%) |
| Hearth beat, mean (std) | 69.68 (11.31) | 69.52 (11.32) | 71.54 (11.08) | 0.001* | 8125 (67.15%) |
| Sleeping hours, mean (std) | 7.18 (0.9) | 7.19 (0.89) | 7.1 (1.11) | 0.055 | 130 (1.07%) |
| Eating habits points, mean (std) | 16.83 (1.99) | 16.84 (1.99) | 16.72 (2.01) | 0.091 |  |
| Exercise habits points, mean (std) | 5.04 (2.13) | 5.04 (2.12) | 5.03 (2.33) | 0.956 | 0 (0.0%) |
| Everyday exercise habits, mean (std) | 2.25 (0.89) | 2.25 (0.89) | 2.34 (0.89) | 0.002* | 181 (1.49%) |
| Aerobic exercise habits, mean (std) | 1.67 (1.04) | 1.68 (1.03) | 1.65 (1.1) | 0.746 | 188 (1.55%) |
| Muscle exercise habits, mean (std) | 1.15 (1.08) | 1.15 (1.08) | 1.07 (1.1) | 0.031* | 293 (2.42%) |
| Smoking, pack-years, mean (std) | 2.97 (7.5) | 2.87 (7.28) | 4.67 (10.25) | 0.001* |  |
| Smoker, n (%) | 3261 (26.95) | 3042 (26.72) | 219 (30.59) | 0.027* | 0 (0.0%) |
| Audit, mean (std) | 4.54 (3.72) | 4.55 (3.71) | 4.25 (3.81) | 0.005* | 0 (0.0%) |
| Anemia, n (%) | 125 (1.03) | 113 (0.99) | 12 (1.68) | 0.085 | 0 (0.0%) |
| Asthma, pulmonary disease, n (%) | 814 (6.73) | 741 (6.51) | 73 (10.2) | <.001** | 0 (0.0%) |
| Diabetes, n (%) | 419 (3.46) | 368 (3.23) | 51 (7.12) | <.001** | 0 (0.0%) |
| Epilepsy, n (%) | 61 (0.5) | 57 (0.5) |  | 0.783 | 0 (0.0%) |
| Skin disease, n (%) | 1305 (10.79) | 1219 (10.71) | 86 (12.01) | 0.291 | 0 (0.0%) |
| Thyroid disorder, n (%) | 520 (4.3) | 466 (4.09) | 54 (7.54) | <.001** | 0 (0.0%) |
| Ear disease, hearing loss, n (%) | 1024 (8.46) | 935 (8.21) | 89 (12.43) | <.001** | 0 (0.0%) |
| Common mental disease, n (%) | 757 (6.26) | 662 (5.82) | 95 (13.27) | <.001** | 0 (0.0%) |
| Migraine, n (%) | 1366 (11.29) | 1252 (11.0) | 114 (15.92) | <.001** | 0 (0.0%) |
| Other illness, n (%) | 1159 (9.58) | 1045 (9.18) | 114 (15.92) | <.001** | 0 (0.0%) |
| Dyspepsia, gastric ulcer, irritable bowel, n (%) | 1807 (14.94) | 1665 (14.63) | 142 (19.83) | <.001** | 0 (0.0%) |
| Hemorrhoides, constipation, n (%) | 1296 (10.71) | 1197 (10.52) | 99 (13.83) | 0.007* | 0 (0.0%) |
| Eye disease, n (%) | 436 (3.6) | 394 (3.46) | 42 (5.87) | 0.002* | 0 (0.0%) |
| Varicose veins, n (%) | 923 (7.63) | 838 (7.36) | 85 (11.87) | <.001** | 0 (0.0%) |
| Cardiovascular disease, n (%) | 624 (5.16) | 556 (4.88) | 68 (9.5) | <.001** | 0 (0.0%) |
| Cancer, n (%) | 244 (2.02) | 208 (1.83) | 36 (5.03) | <.001** | 0 (0.0%) |
| Musculoskeletal disease, n (%) | 1574 (13.01) | 1373 (12.06) | 201 (28.07) | <.001** | 0 (0.0%) |
| Insomnia, tiredness, n (%) | 1828 (15.11) | 1663 (14.61) | 165 (23.04) | <.001** | 0 (0.0%) |
| Hypertension, n (%) | 1508 (12.46) | 1379 (12.11) | 129 (18.02) | <.001** | 0 (0.0%) |
| Number of diseases, mean (std) | 1.47 (1.6) | 1.42 (1.55) | 2.32 (1.96) | <.001** | 0 (0.0%) |
| Excema symptom, n (%) | 2592 (21.42) | 2436 (21.4) | 156 (21.79) | 0.814 | 0 (0.0%) |
| Cough, rhinitis symptom, n (%) | 5110 (42.23) | 4797 (42.14) | 313 (43.72) | 0.413 | 0 (0.0%) |
| Gastrointestinal symptom, n (%) | 2375 (19.63) | 2209 (19.41) | 166 (23.18) | 0.015* | 0 (0.0%) |
| Dizziness symptom, n (%) | 1844 (15.24) | 1670 (14.67) | 174 (24.3) | <.001** | 0 (0.0%) |
| Fainting, conciousness disturbances symptom, n (%) | 171 (1.41) | 148 (1.3) | 23 (3.21) | <.001** | 0 (0.0%) |
| Arrhythmia symptom, n (%) | 901 (7.45) | 821 (7.21) | 80 (11.17) | <.001** | 0 (0.0%) |
| Anxiety or panic symptoms, n (%) | 612 (5.06) | 540 (4.74) | 72 (10.06) | <.001** | 0 (0.0%) |
| Gloomiess, irritability, tearfulness symptom, n (%) | 1975 (16.32) | 1796 (15.78) | 179 (25.0) | <.001** | 0 (0.0%) |
| Joylessness, hopelessness symptom, n (%) | 1170 (9.67) | 1066 (9.36) | 104 (14.53) | <.001** | 0 (0.0%) |
| Problem of sex life symptom, n (%) | 657 (5.43) | 605 (5.31) | 52 (7.26) | 0.033* | 0 (0.0%) |
| Memory problems symptom, n (%) | 1287 (10.64) | 1164 (10.23) | 123 (17.18) | <.001** | 0 (0.0%) |
| Concentration difficulties symptom, n (%) | 1369 (11.31) | 1249 (10.97) | 120 (16.76) | <.001** | 0 (0.0%) |
| Learning difficulties symptom, n (%) | 626 (5.17) | 562 (4.94) | 64 (8.94) | <.001** | 0 (0.0%) |
| Vision impairment symptom, n (%) | 1510 (12.48) | 1384 (12.16) | 126 (17.6) | <.001** | 0 (0.0%) |
| Hearing impairment symptom, n (%) | 938 (7.75) | 857 (7.53) | 81 (11.31) | <.001** | 0 (0.0%) |
| Weakness or clumsiness of limbs symptom, n (%) | 906 (7.49) | 768 (6.75) | 138 (19.27) | <.001** | 0 (0.0%) |
| Headache symptom, n (%) | 3041 (25.13) | 2830 (24.86) | 211 (29.47) | 0.007* | 0 (0.0%) |
| Pain symptom, n (%) | 3156 (26.08) | 2838 (24.93) | 318 (44.41) | <.001** | 0 (0.0%) |
| Musculoskeletal symptom, n (%) | 4429 (36.61) | 4055 (35.62) | 374 (52.23) | <.001** | 0 (0.0%) |
| Number of symptoms, mean (std) | 2.87 (2.78) | 2.79 (2.73) | 4.01 (3.27) | <.001** | 0 (0.0%) |
| Suffered from insomnia, mean (std) | 0.45 (0.64) | 0.44 (0.63) | 0.63 (0.74) | <.001** | 90 (0.74%) |
| Felt melancholic, mean (std) | 0.25 (0.51) | 0.24 (0.49) | 0.4 (0.66) | <.001** | 233 (1.92%) |
| Felt everything was an effort, mean (std) | 0.27 (0.53) | 0.26 (0.51) | 0.47 (0.69) | <.001** | 120 (0.99%) |
| Felt unenergetic, mean (std) | 0.36 (0.58) | 0.35 (0.57) | 0.54 (0.72) | <.001** | 125 (1.03%) |
| Felt lonely, mean (std) | 0.18 (0.46) | 0.17 (0.45) | 0.24 (0.55) | <.001** | 102 (0.84%) |
| Felt hopeless about the future, mean (std) | 0.15 (0.41) | 0.14 (0.4) | 0.28 (0.58) | <.001** | 130 (1.07%) |
| Felt impossible to find pleasure in life, mean (std) | 0.14 (0.39) | 0.13 (0.37) | 0.25 (0.53) | <.001** | 124 (1.02%) |
| Had feelings of worthlessness, mean (std) | 0.13 (0.38) | 0.12 (0.37) | 0.2 (0.5) | <.001** | 124 (1.02%) |
| Felt all joy has gone from life, mean (std) | 0.11 (0.35) | 0.1 (0.34) | 0.22 (0.49) | <.001** | 144 (1.19%) |
| Felt that even help from my family and friends cannot ease my melancholy, mean (std) | 0.09 (0.34) | 0.09 (0.33) | 0.17 (0.47) | <.001** | 173 (1.43%) |
| Do you feel drained of energy by your work, mean (std) | 0.34 (0.61) | 0.33 (0.6) | 0.52 (0.75) | <.001** | 138 (1.14%) |
| Felt mental stress, mean (std) | 0.48 (0.65) | 0.47 (0.64) | 0.63 (0.74) | <.001** | 162 (1.33%) |
| During past 1 mo often bothered by feeling down, depressed or hopeless, n (%) | 1502 (12.41) | 1374 (12.07) | 128 (17.88) | <.001** | 0 (0.0%) |
| During past 1 mo often bothered by little interest or pleasure in doing things, n (%) | 1275 (10.54) | 1151 (10.11) | 124 (17.32) | <.001** | 0 (0.0%) |
| Deps score, mean (std) | 2.11 (3.17) | 2.03 (3.05) | 3.32 (4.49) | <.001** |  |
| I enjoy my job, mean (std) | 0.42 (0.63) | 0.42 (0.63) | 0.46 (0.68) | 0.228 | 121 (1.0%) |
| The mental workload of my job is suitable for me, mean (std) | 0.59 (0.71) | 0.58 (0.7) | 0.68 (0.76) | 0.001* | 192 (1.58%) |
| I am familiar with the expectations and duties of my work tasks, mean (std) | 0.32 (0.57) | 0.32 (0.57) | 0.35 (0.61) | 0.411 | 141 (1.16%) |
| My capabilities are proportional with my job´s demands, mean (std) | 0.4 (0.61) | 0.4 (0.61) | 0.41 (0.64) | 0.932 | 142 (1.17%) |
| My work is appreciated, and I am praised for a job well done, mean (std) | 0.96 (0.86) | 0.95 (0.86) | 1.1 (0.9) | <.001** | 156 (1.28%) |
| My job is versatile, mean (std) | 0.69 (0.82) | 0.68 (0.81) | 0.82 (0.91) | <.001** | 174 (1.43%) |
| I can influence the contents, pace, and hours in my job, mean (std) | 1.04 (0.97) | 1.02 (0.96) | 1.36 (1.06) | <.001** | 137 (1.13%) |
| I have the possibility of self-reformation at my job, mean (std) | 0.85 (0.88) | 0.83 (0.87) | 1.07 (0.97) | <.001** | 171 (1.41%) |
| I can rely on the continuance of my employment, mean (std) | 0.86 (0.94) | 0.85 (0.94) | 0.95 (1.01) | 0.024* | 178 (1.47%) |
| I face a threat of violence in my job, mean (std) | 0.2 (0.61) | 0.2 (0.6) | 0.28 (0.71) | <.001** | 137 (1.13%) |
| Does your symptom hinder coping at work, n (%) | 1769 (14.62) | 1548 (13.6) | 221 (30.87) | <.001** | 0 (0.0%) |
| Does your disease hinder coping at work, n (%) | 1112 (9.19) | 942 (8.28) | 170 (23.74) | <.001** | 0 (0.0%) |
| Personal prognosis of work ability in current job 2 years from now, mean (std) | 0.1 (0.33) | 0.09 (0.31) | 0.25 (0.52) | <.001** | 0 (0.0%) |
| Do problems of sleep, alertness, stress or mood hinder coping at work, n (%) | 1375 (11.36) | 1226 (10.77) | 149 (20.81) | <.001** | 0 (0.0%) |
| Work ability has become worse, n (%) | 2165 (18.07) | 1936 (17.16) | 229 (32.67) | <.001** | 119 (0.98%) |
| Self-rated change in work ability, mean (std) | 1.1 (0.5) | 1.09 (0.5) | 1.25 (0.59) | <.001** | 119 (0.98%) |
| Self-rated work ability, mean (std) | 1.74 (1.32) | 1.7 (1.27) | 2.46 (1.81) | <.001** | 83 (0.68%) |
| Work ability has improved, n (%) | 10990 (91.74) | 10345 (91.72) | 645 (92.01) | 0.463 | 119 (0.98%) |
| Work ability has not changed, n (%) | 8825 (73.66) | 8409 (74.55) | 416 (59.34) | <.001** | 119 (0.98%) |
| The physical demands of my job are suitable for me, mean (std) | 0.4 (0.67) | 0.39 (0.66) | 0.7 (0.81) | <.001** | 154 (1.27%) |
| How well do you recover from work, mean (std) | 0.48 (0.56) | 0.47 (0.55) | 0.69 (0.65) | <.001** | 157 (1.29%) |
| Management provides the necessary help and encouragement, mean (std) | 0.82 (0.85) | 0.81 (0.84) | 0.96 (0.92) | <.001** | 180 (1.48%) |
| Managerial work is fair and equal, mean (std) | 0.85 (0.88) | 0.84 (0.87) | 1.09 (0.96) | <.001** | 201 (1.66%) |
| There is a good spirit within our personnel, mean (std) | 0.76 (0.8) | 0.75 (0.79) | 0.83 (0.83) | 0.015* | 170 (1.40%) |
| I have not witnessed bullying or harassment at our workplace, mean (std) | 0.7 (0.91) | 0.69 (0.9) | 0.85 (0.98) | <.001** | 160 (1.32%) |

1. Troyanskaya O, Cantor M, Sherlock G, Brown P, Hastie T, Tibshirani R, ym. Missing value estimation methods for DNA microarrays. Bioinformatics. 2001;17(6):520–5.

## Table S2. Descriptive statistics of background variables and their distribution in the different clusters.

Clusters are named: C1 Healthy employees; C2 Managerial performance and workplace atmosphere; C3 Mood and depression; C4 Cardiovascular diseases; C5 Dizziness and sensory symptoms; C6 Work ability

|  | **Item name** | **All** | **C1** | **C2** | **C3** | **C4** | **C5** | **C6** |
| --- | --- | --- | --- | --- | --- | --- | --- | --- |
|  | Employees, n | 12099 | 5503 | 2863 | 800 | 989 | 543 | 1401 |
| Demography | Gender, n (%) | 5163 (42.67) | 2265 (41.16) | 1244 (43.45) | 390 (48.75) | 293 (29.63) | 295 (54.33) | 676 (48.25) |
|  | Age, mean (std) | 42.69 (11.45) | 40.78 (11.33) | 40.85 (10.64) | 42.55 (11.01) | 53.05 (7.95) | 43.42 (11.25) | 46.4 (11.12) |
| Job description | Supervisor, n (%) | 2046 (16.91) | 1219 (22.15) | 222 (7.75) | 113 (14.12) | 190 (19.21) | 51 (9.39) | 251 (17.92) |
|  | White-collar worker, n (%) | 5231 (43.23) | 2731 (49.63) | 1003 (35.03) | 336 (42.0) | 457 (46.21) | 231 (42.54) | 473 (33.76) |
|  | Blue-collar worker, n (%) | 6484 (53.59) | 2605 (47.34) | 1784 (62.31) | 439 (54.87) | 487 (49.24) | 296 (54.51) | 873 (62.31) |
|  | Shift work, n (%) | 2871 (23.73) | 1201 (21.82) | 777 (27.14) | 204 (25.5) | 191 (19.31) | 122 (22.47) | 376 (26.84) |
|  | Night work, n (%) | 740 (6.12) | 238 (4.32) | 293 (10.23) | 50 (6.25) | 42 (4.25) | 43 (7.92) | 74 (5.28) |
|  | Part-time work, n (%) | 1603 (13.25) | 764 (13.88) | 289 (10.09) | 138 (17.25) | 82 (8.29) | 82 (15.1) | 248 (17.7) |
| Health habits | BMI, mean (std) | 26.13 (4.52) | 25.51 (4.15) | 26.01 (4.34) | 26.64 (5.07) | 29.38 (5.28) | 25.84 (4.57) | 26.33 (4.33) |
|  | Blood pressure elevated, n (%) | 1332 (11.01) | 393 (7.14) | 229 (8.0) | 123 (15.38) | 354 (35.79) | 60 (11.05) | 173 (12.35) |
|  | Cholesterol elevated, n (%) | 1961 (16.21) | 713 (12.96) | 344 (12.02) | 153 (19.12) | 380 (38.42) | 83 (15.29) | 288 (20.56) |
|  | Blood glucose elevated, n (%) | 461 (3.81) | 18 (0.33) | 17 (0.59) | 35 (4.38) | 349 (35.29) | 14 (2.58) | 28 (2.0) |
|  | Sleeping hours, mean (std) | 6.99 (0.93) | 7.08 (0.84) | 6.93 (0.91) | 6.86 (1.23) | 7.05 (0.96) | 6.9 (1.04) | 6.86 (1.02) |
|  | Eating habits points, mean (std) | 16.83 (1.99) | 17.05 (1.85) | 16.78 (2.0) | 16.22 (2.24) | 16.52 (2.08) | 16.69 (2.14) | 16.7 (2.07) |
|  | Excercise habits points, mean (std) | 5.04 (2.13) | 5.21 (2.11) | 5.14 (2.15) | 4.41 (2.19) | 4.6 (2.11) | 4.85 (2.06) | 4.91 (2.1) |
|  | Everyday exercise habits, mean (std) | 2.23 (0.9) | 2.25 (0.89) | 2.23 (0.91) | 2.1 (0.97) | 2.13 (0.94) | 2.22 (0.9) | 2.32 (0.86) |
|  | Aerobic exercise habits, mean (std) | 1.65 (1.05) | 1.71 (1.02) | 1.69 (1.04) | 1.4 (1.09) | 1.6 (1.06) | 1.55 (1.05) | 1.55 (1.06) |
|  | Muscle exercise habits, mean (std) | 1.12 (1.08) | 1.22 (1.08) | 1.18 (1.11) | 0.88 (1.05) | 0.84 (0.99) | 1.03 (1.04) | 0.99 (1.06) |
|  | Smoker, n (%) | 3261 (26.95) | 1450 (26.35) | 733 (25.6) | 277 (34.62) | 217 (21.94) | 159 (29.28) | 425 (30.34) |
|  | Audit, mean (std) | 4.54 (3.72) | 4.3 (3.31) | 4.59 (3.62) | 5.81 (5.17) | 4.97 (4.23) | 4.67 (4.36) | 4.28 (3.58) |
| Diseases | Asthma, pulmonary disease, n (%) | 814 (6.73) | 326 (5.92) | 164 (5.73) | 65 (8.12) | 90 (9.1) | 63 (11.6) | 106 (7.57) |
|  | Diabetes, n (%) | 419 (3.46) | 9 (0.16) | 12 (0.42) | 21 (2.62) | 348 (35.19) | 7 (1.29) | 22 (1.57) |
|  | Common mental disease, n (%) | 757 (6.26) | 172 (3.13) | 88 (3.07) | 269 (33.62) | 59 (5.97) | 70 (12.89) | 99 (7.07) |
|  | Cardiovascular disease, n (%) | 624 (5.16) | 104 (1.89) | 99 (3.46) | 51 (6.38) | 208 (21.03) | 84 (15.47) | 78 (5.57) |
|  | Cancer, n (%) | 244 (2.02) | 58 (1.05) | 51 (1.78) | 16 (2.0) | 65 (6.57) | 12 (2.21) | 42 (3.0) |
|  | Musculoskeletal disease, n (%) | 1574 (13.01) | 374 (6.8) | 239 (8.35) | 113 (14.12) | 159 (16.08) | 95 (17.5) | 594 (42.4) |
|  | Insomnia, tiredness, n (%) | 1828 (15.11) | 435 (7.9) | 333 (11.63) | 359 (44.88) | 117 (11.83) | 183 (33.7) | 401 (28.62) |
|  | Hypertension, n (%) | 1508 (12.46) | 291 (5.29) | 189 (6.6) | 121 (15.12) | 614 (62.08) | 59 (10.87) | 234 (16.7) |
|  | Number of diseases, mean (std) | 1.47 (1.6) | 0.95 (1.16) | 1.1 (1.26) | 2.39 (1.89) | 2.97 (1.94) | 2.53 (1.96) | 2.26 (1.68) |
|  | Excema symptom, n (%) | 2592 (21.42) | 994 (18.06) | 661 (23.09) | 200 (25.0) | 148 (14.96) | 216 (39.78) | 373 (26.62) |
|  | Cough, rhinitis symptom, n (%) | 5110 (42.23) | 2124 (38.6) | 1262 (44.08) | 386 (48.25) | 293 (29.63) | 394 (72.56) | 651 (46.47) |
|  | Gastrointestinal symptom, n (%) | 2375 (19.63) | 814 (14.79) | 526 (18.37) | 239 (29.88) | 137 (13.85) | 320 (58.93) | 339 (24.2) |
|  | Dizziness symptom, n (%) | 1844 (15.24) | 493 (8.96) | 369 (12.89) | 216 (27.0) | 107 (10.82) | 364 (67.03) | 295 (21.06) |
|  | Fainting, conciousness disturbances symptom, n (%) | 171 (1.41) |  |  | 10 (1.25) |  | 142 (26.15) | 11 (0.79) |
|  | Arrhythmia symptom, n (%) | 901 (7.45) | 184 (3.34) | 171 (5.97) | 90 (11.25) | 124 (12.54) | 201 (37.02) | 131 (9.35) |
|  | Anxiety or panic symptoms, n (%) | 612 (5.06) | 112 (2.04) | 55 (1.92) | 228 (28.5) | 15 (1.52) | 137 (25.23) | 65 (4.64) |
|  | Gloomiess, irritability, tearfulness symptom, n (%) | 1975 (16.32) | 452 (8.21) | 344 (12.02) | 586 (73.25) | 48 (4.85) | 246 (45.3) | 299 (21.34) |
|  | Joylessness, hopelessness symptom, n (%) | 1170 (9.67) | 148 (2.69) | 144 (5.03) | 550 (68.75) | 38 (3.84) | 158 (29.1) | 132 (9.42) |
|  | Problem of sex life symptom, n (%) | 657 (5.43) | 141 (2.56) | 63 (2.2) | 179 (22.38) | 90 (9.1) | 121 (22.28) | 63 (4.5) |
|  | Memory problems symptom, n (%) | 1287 (10.64) | 204 (3.71) | 157 (5.48) | 231 (28.88) | 100 (10.11) | 325 (59.85) | 270 (19.27) |
|  | Concentration difficulties symptom, n (%) | 1369 (11.31) | 209 (3.8) | 163 (5.69) | 362 (45.25) | 54 (5.46) | 278 (51.2) | 303 (21.63) |
|  | Learning difficulties symptom, n (%) | 626 (5.17) | 42 (0.76) | 46 (1.61) | 155 (19.38) | 42 (4.25) | 203 (37.38) | 138 (9.85) |
|  | Vision impairment symptom, n (%) | 1510 (12.48) | 424 (7.7) | 296 (10.34) | 117 (14.62) | 167 (16.89) | 269 (49.54) | 237 (16.92) |
|  | Hearing impairment symptom, n (%) | 938 (7.75) | 184 (3.34) | 121 (4.23) | 76 (9.5) | 173 (17.49) | 235 (43.28) | 149 (10.64) |
|  | Weakness or clumsiness of limbs symptom, n (%) | 906 (7.49) | 119 (2.16) | 73 (2.55) | 114 (14.25) | 95 (9.61) | 198 (36.46) | 307 (21.91) |
|  | Headache symptom, n (%) | 3041 (25.13) | 1182 (21.48) | 757 (26.44) | 247 (30.88) | 74 (7.48) | 341 (62.8) | 440 (31.41) |
|  | Pain symptom, n (%) | 3156 (26.08) | 899 (16.34) | 663 (23.16) | 256 (32.0) | 212 (21.44) | 318 (58.56) | 808 (57.67) |
|  | Musculoskeletal symptom, n (%) | 4429 (36.61) | 1471 (26.73) | 942 (32.9) | 303 (37.88) | 387 (39.13) | 308 (56.72) | 1018 (72.66) |
|  | Number of symptoms, mean (std) | 2.87 (2.78) | 1.85 (1.73) | 2.38 (1.99) | 5.68 (3.3) | 2.33 (2.21) | 8.79 (3.69) | 4.3 (2.62) |
| Depression questions | Suffered from insomnia, mean (std) | 0.45 (0.64) | 0.32 (0.52) | 0.43 (0.58) | 1.0 (0.84) | 0.39 (0.58) | 0.66 (0.71) | 0.68 (0.75) |
|  | Felt melancolic, mean (std) | 0.25 (0.5) | 0.11 (0.32) | 0.17 (0.39) | 1.32 (0.65) | 0.16 (0.38) | 0.42 (0.57) | 0.3 (0.49) |
|  | Felt everything was an effort, mean (std) | 0.27 (0.53) | 0.11 (0.32) | 0.2 (0.42) | 1.22 (0.74) | 0.2 (0.42) | 0.5 (0.62) | 0.48 (0.61) |
|  | Felt unenergetic, mean (std) | 0.36 (0.58) | 0.19 (0.43) | 0.31 (0.5) | 1.26 (0.71) | 0.32 (0.53) | 0.6 (0.64) | 0.58 (0.62) |
|  | Felt lonely, mean (std) | 0.18 (0.45) | 0.09 (0.31) | 0.13 (0.35) | 0.98 (0.83) | 0.14 (0.4) | 0.24 (0.49) | 0.15 (0.38) |
|  | Felt hopeless about the future, mean (std) | 0.15 (0.41) | 0.04 (0.2) | 0.11 (0.33) | 0.97 (0.71) | 0.1 (0.33) | 0.22 (0.48) | 0.18 (0.42) |
|  | Felt impossible to find pleasure in life, mean (std) | 0.13 (0.38) | 0.03 (0.18) | 0.08 (0.27) | 1.0 (0.63) | 0.08 (0.29) | 0.2 (0.44) | 0.17 (0.39) |
|  | Had feelings of worthlessness, mean (std) | 0.13 (0.38) | 0.03 (0.17) | 0.1 (0.32) | 0.84 (0.71) | 0.09 (0.3) | 0.22 (0.46) | 0.13 (0.36) |
|  | Felt all joy has gone from life, mean (std) | 0.11 (0.35) | 0.02 (0.15) | 0.05 (0.22) | 0.95 (0.66) | 0.07 (0.26) | 0.17 (0.38) | 0.1 (0.31) |
|  | Felt that even help from my family and friends cannot ease my melancholy, mean (std) | 0.09 (0.34) | 0.02 (0.14) | 0.03 (0.18) | 0.91 (0.68) | 0.04 (0.21) | 0.14 (0.39) | 0.07 (0.27) |
|  | Do you feel drained of energy by your work, mean (std) | 0.34 (0.61) | 0.14 (0.36) | 0.36 (0.58) | 1.01 (0.9) | 0.27 (0.52) | 0.4 (0.63) | 0.7 (0.79) |
|  | Felt mental stress, mean (std) | 0.47 (0.65) | 0.29 (0.49) | 0.44 (0.59) | 1.34 (0.84) | 0.36 (0.54) | 0.69 (0.71) | 0.73 (0.73) |
| Psychosocial questions | I enjoy my job, mean (std) | 0.42 (0.63) | 0.13 (0.35) | 0.82 (0.7) | 0.75 (0.79) | 0.24 (0.48) | 0.48 (0.64) | 0.6 (0.7) |
|  | The mental workload of my job is suitable for me, mean (std) | 0.58 (0.7) | 0.27 (0.48) | 0.87 (0.71) | 1.11 (0.84) | 0.49 (0.61) | 0.72 (0.74) | 0.92 (0.79) |
|  | I am familiar with the expectations and duties of my work tasks, mean (std) | 0.32 (0.57) | 0.16 (0.39) | 0.54 (0.69) | 0.58 (0.73) | 0.19 (0.43) | 0.38 (0.6) | 0.4 (0.63) |
|  | My capabilities are proportional with my job´s demands, mean (std) | 0.4 (0.61) | 0.23 (0.45) | 0.61 (0.73) | 0.64 (0.76) | 0.3 (0.52) | 0.47 (0.64) | 0.49 (0.66) |
|  | My work is appreciated, and I am praised for a job well done, mean (std) | 0.95 (0.86) | 0.5 (0.59) | 1.63 (0.77) | 1.33 (0.91) | 0.91 (0.78) | 1.07 (0.87) | 1.15 (0.88) |
|  | My job is versatile, mean (std) | 0.68 (0.82) | 0.38 (0.59) | 1.17 (0.88) | 0.94 (0.93) | 0.55 (0.71) | 0.84 (0.85) | 0.77 (0.88) |
|  | I can influence the contents, pace, and hours in my job, mean (std) | 1.04 (0.96) | 0.67 (0.78) | 1.59 (0.94) | 1.34 (0.99) | 0.89 (0.93) | 1.17 (0.94) | 1.27 (1.0) |
|  | I have the possibility of self-reformation at my job, mean (std) | 0.84 (0.87) | 0.43 (0.61) | 1.44 (0.87) | 1.16 (0.94) | 0.77 (0.79) | 0.96 (0.86) | 1.04 (0.93) |
|  | I can rely on the continuance of my employment, mean (std) | 0.85 (0.94) | 0.5 (0.73) | 1.38 (0.98) | 1.07 (0.98) | 0.86 (0.93) | 1.05 (1.0) | 0.93 (0.95) |
|  | I face a threat of violence in my job, mean (std) | 0.2 (0.61) | 0.19 (0.59) | 0.16 (0.54) | 0.26 (0.64) | 0.22 (0.67) | 0.25 (0.66) | 0.28 (0.7) |
| Work ability questions | Does your symptom hinder coping at work, n (%) | 1769 (14.62) | 220 (4.0) | 220 (7.68) | 264 (33.0) | 106 (10.72) | 128 (23.57) | 831 (59.31) |
|  | Does your disease hinder coping at work, n (%) | 1112 (9.19) | 88 (1.6) | 86 (3.0) | 189 (23.62) | 63 (6.37) | 78 (14.36) | 608 (43.4) |
|  | Personal prognosis of work ability in current job 2 years from now, mean (std) | 0.1 (0.33) | 0.01 (0.11) | 0.05 (0.23) | 0.26 (0.51) | 0.12 (0.35) | 0.09 (0.33) | 0.42 (0.62) |
|  | Do problems of sleep, alertness, stress or mood hinder coping at work, n (%) | 1375 (11.36) | 164 (2.98) | 222 (7.75) | 356 (44.5) | 67 (6.77) | 103 (18.97) | 463 (33.05) |
|  | Work ability has become worse, n (%) | 2167 (17.91) | 175 (3.18) | 218 (7.61) | 315 (39.38) | 137 (13.85) | 147 (27.07) | 1175 (83.87) |
|  | Self-rated change in work ability, mean (std) | 1.1 (0.5) | 0.93 (0.36) | 1.01 (0.38) | 1.31 (0.62) | 1.01 (0.52) | 1.19 (0.56) | 1.83 (0.4) |
|  | Self-rated work ability, mean (std) | 1.74 (1.32) | 1.2 (0.87) | 1.68 (1.04) | 2.83 (1.55) | 1.69 (1.12) | 2.11 (1.3) | 3.27 (1.62) |
|  | Work ability has improved, n (%) | 11080 (91.58) | 4931 (89.61) | 2675 (93.43) | 732 (91.5) | 857 (86.65) | 498 (91.71) | 1387 (99.0) |
|  | Work ability has not changed, n (%) | 8873 (73.34) | 4739 (86.12) | 2447 (85.47) | 415 (51.88) | 719 (72.7) | 350 (64.46) | 203 (14.49) |
|  | The physical demands of my job are suitable for me, mean (std) | 0.4 (0.67) | 0.19 (0.47) | 0.54 (0.7) | 0.56 (0.75) | 0.33 (0.59) | 0.45 (0.64) | 0.9 (0.87) |
|  | How well do you recover from work, mean (std) | 0.48 (0.56) | 0.28 (0.45) | 0.53 (0.53) | 0.91 (0.61) | 0.43 (0.52) | 0.64 (0.58) | 0.86 (0.6) |
| Supervisor and atmosphere questions | Management provides the necessary help and encouragement, mean (std) | 0.81 (0.85) | 0.38 (0.54) | 1.48 (0.82) | 1.12 (0.94) | 0.81 (0.8) | 0.86 (0.85) | 0.97 (0.86) |
|  | Managerial work is fair and equal, mean (std) | 0.84 (0.88) | 0.39 (0.57) | 1.53 (0.84) | 1.08 (0.96) | 0.84 (0.83) | 1.01 (0.91) | 1.02 (0.9) |
|  | There is a good spirit within our personnel, mean (std) | 0.75 (0.8) | 0.41 (0.58) | 1.26 (0.81) | 1.03 (0.89) | 0.74 (0.75) | 0.77 (0.82) | 0.87 (0.81) |
|  | I have not witnessed bullying or harassment at our workplace, mean (std) | 0.69 (0.9) | 0.34 (0.64) | 1.19 (0.98) | 1.01 (1.02) | 0.67 (0.88) | 0.84 (0.97) | 0.84 (0.94) |
| Sickness absences | SA short period (next 2 years), n (%) | 1055 (8.72) | 366 (6.65) | 296 (10.34) | 84 (10.5) | 70 (7.08) | 75 (13.81) | 164 (11.71) |
|  | SA long period (next 2 years), n (%) | 716 (5.92) | 199 (3.62) | 137 (4.79) | 75 (9.38) | 79 (7.99) | 44 (8.1) | 182 (12.99) |


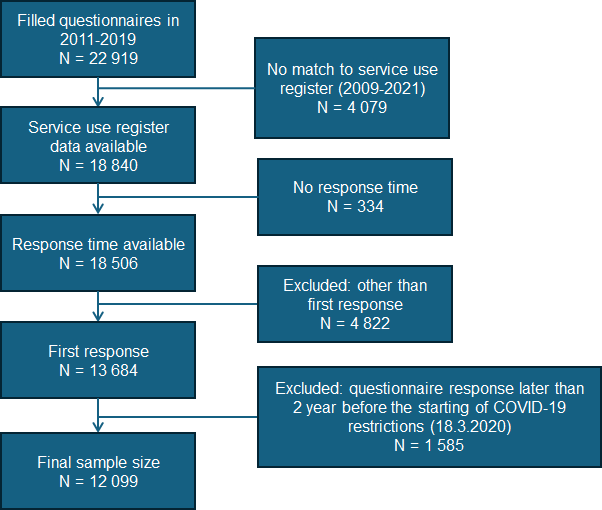


Figure S1. Study flow. For the study we selected employees who completed the occupational health care questionnaire at least once during the years 2011-2019 and whose questionnaires could be linked to service usage data. Employees whose responses were later than two years before the start of COVID-19 were excluded from the study. Furthermore, employees with no response time available were excluded. The final study sample consisted of 12 099 employees.


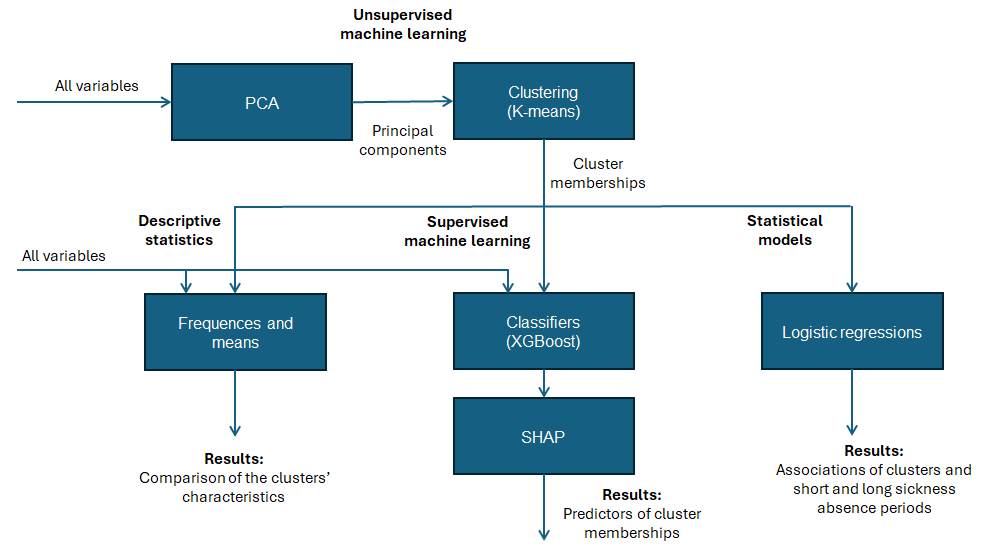


Figure S2. Analytical pipeline. In the first step, the latent dimensions are defined for the employee characteristics by principal component analysis (PCA). In the second step, the clusters are calculated from datapoints expressed by the latent dimensions using the K-means algorithm. The resulting clusters are analyzed by three methods: Descriptive statistics compare the characteristics of the employees in different clusters, supervised machine learning methods (XGBoost and Shapley (SHAP) values) are used to identify the variables that predict the memberships of different clusters and statistical models are used to assess the associations of the clusters for long and short sickness absence (SA) episodes.


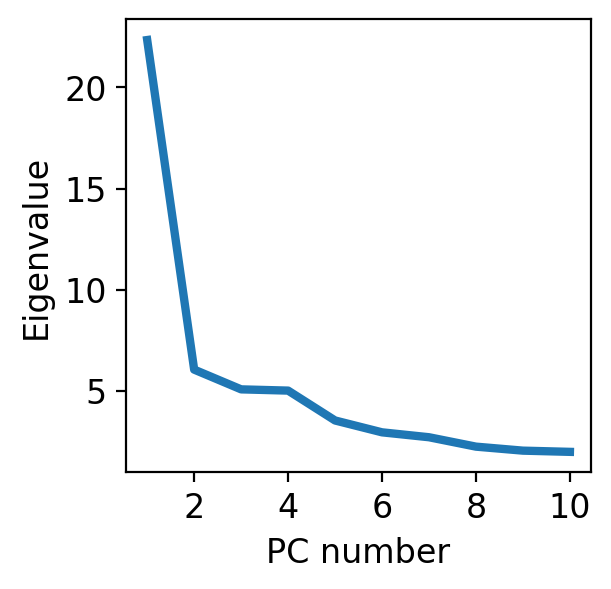


Figure S3. Eigenvalues for the 10 first principal components of the data set. The principal components are sorted in decreasing order of eigenvalues. Eigenvalues correspond to the amount of the variation explained by each component.


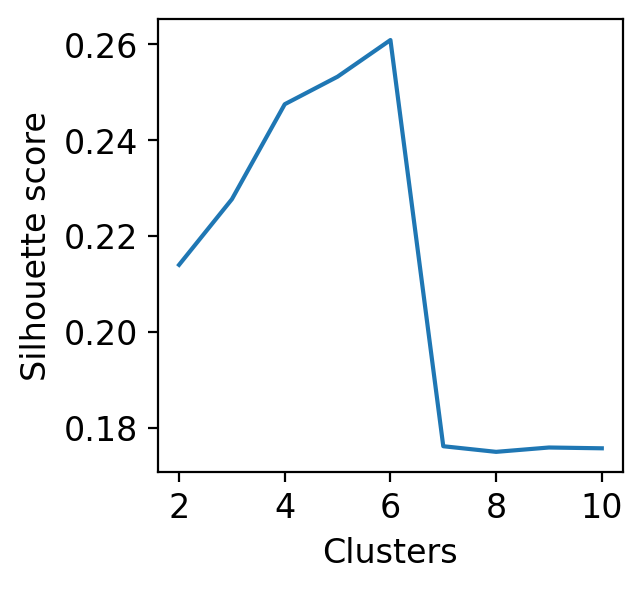


Figure S4. Silhouette score for the clustering solutions from 2 to 10 clusters.

**
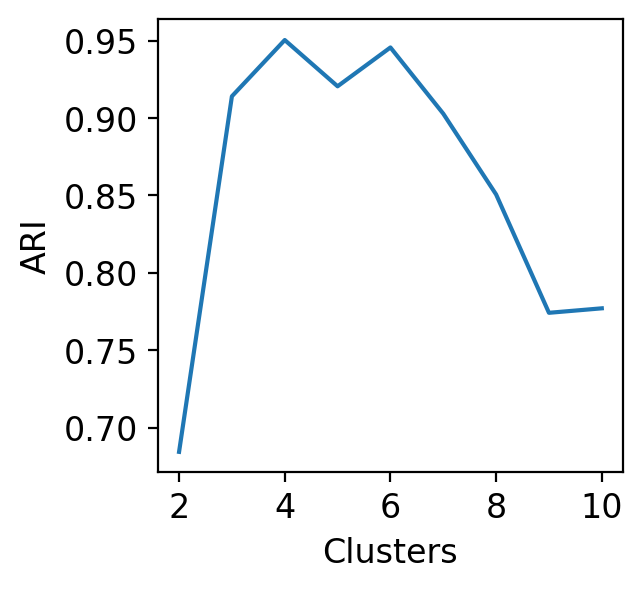
**

## Figure S5. Adjusted Rand Index (ARI) for the clustering solutions from 2 to 10 clusters.


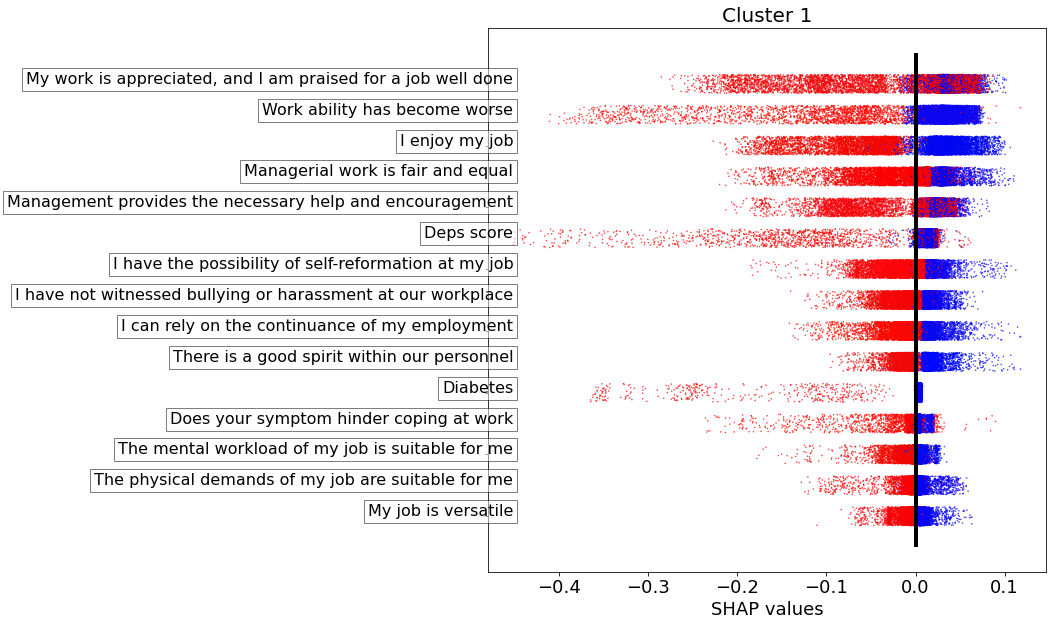


Figure S6. Shapley values (SHAP) for the 15 most important variables predicting cluster membership of Cluster 1 (Healthy employees). Shapley values were calculated from the supervised classification model (XGBoost) that was trained for discriminating cluster 1 from all other clusters. The red points indicate higher employee-specific variable values than the average value of the variable and the blue points are lower employee-specific variable values than the average value of the variable. A longer distance between red and blue points indicates a higher capacity of a variable to change predicted cluster membership. The points at the right side of the black vertical line increase the probability of cluster membership. The points at the left side decrease the probability.


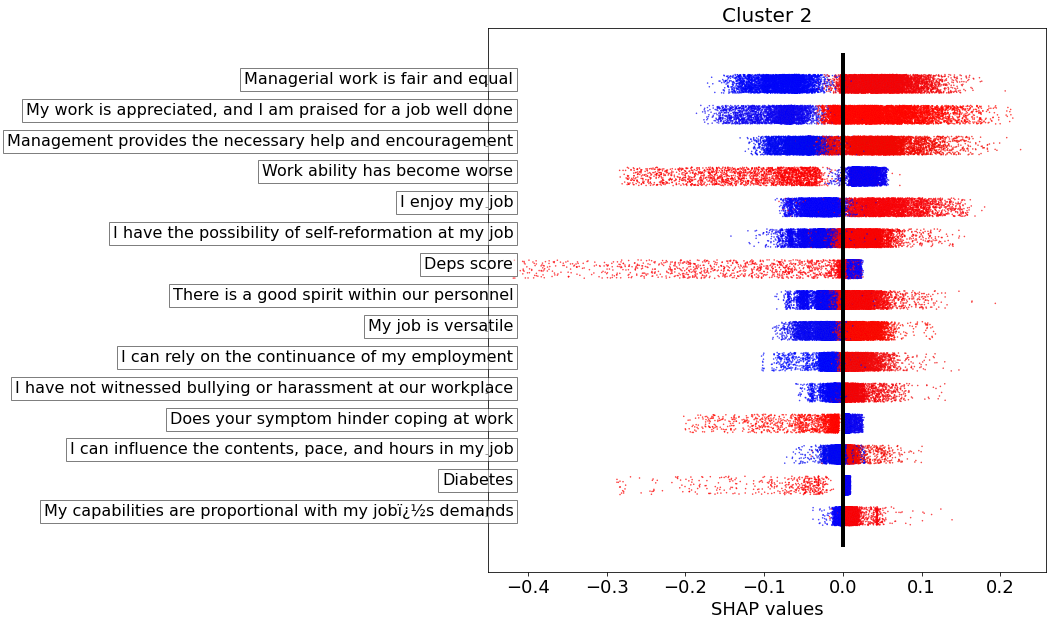


Figure S7. Shapley values (SHAP) for the 15 most important variables predicting cluster membership of Cluster 2 (Managerial performance and workplace atmosphere). Shapley values were calculated from the supervised classification model (XGBoost) that was trained for discriminating cluster 2 from all other clusters. The red points indicate higher employee-specific variable values than the average value of the variable and the blue points are lower employee-specific variable values than the average value of the variable. A longer distance between red and blue points indicates a higher capacity of a variable to change predicted cluster membership. The points at the right side of the black vertical line increase the probability of cluster membership. The points at the left side decrease the probability.


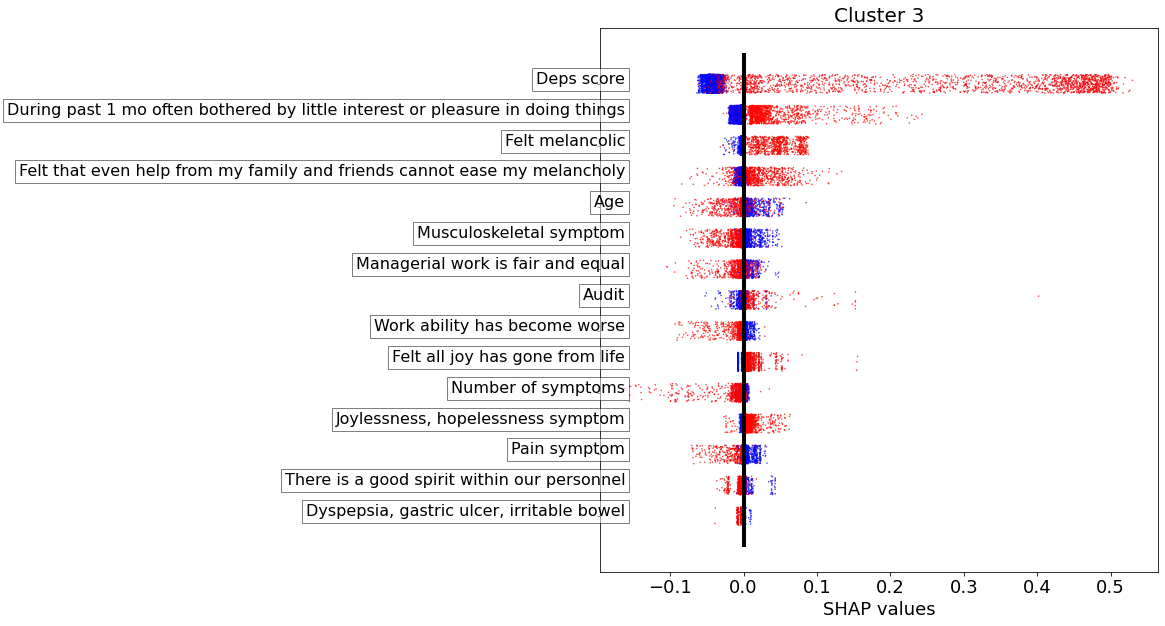


Figure S8. Shapley values (SHAP) for the 15 most important variables predicting cluster membership of Cluster 3 (Mood and depression). Shapley values were calculated from the supervised classification model (XGBoost) that was trained for discriminating cluster 3 from all other clusters. The red points indicate higher employee-specific variable values than the average value of the variable and the blue points are lower employee-specific variable values than the average value of the variable. A longer distance between red and blue points indicates a higher capacity of a variable to change predicted cluster membership. The points at the right side of the black vertical line increase the probability of cluster membership. The points at the left side decrease the probability.


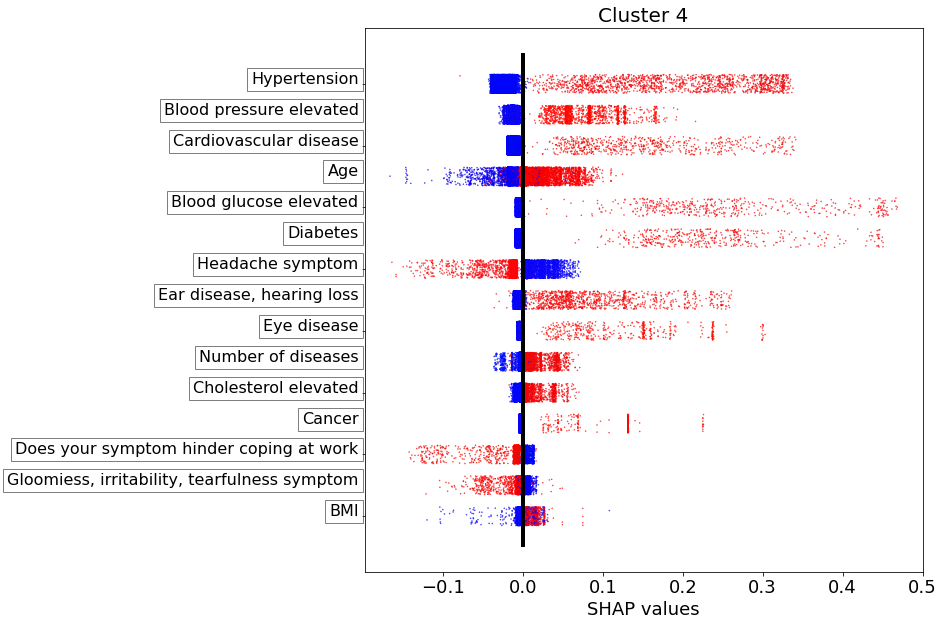


Figure S9. Shapley values (SHAP) for the 15 most important variables predicting cluster membership of Cluster 4 (Cardiovascular diseases). Shapley values were calculated from the supervised classification model (XGBoost) that was trained for discriminating cluster 4 from all other clusters. The red points indicate higher employee-specific variable values than the average value of the variable and the blue points are lower employee-specific variable values than the average value of the variable. A longer distance between red and blue points indicates a higher capacity of a variable to change predicted cluster membership. The points at the right side of the black vertical line increase the probability of cluster membership. The points at the left side decrease the probability.


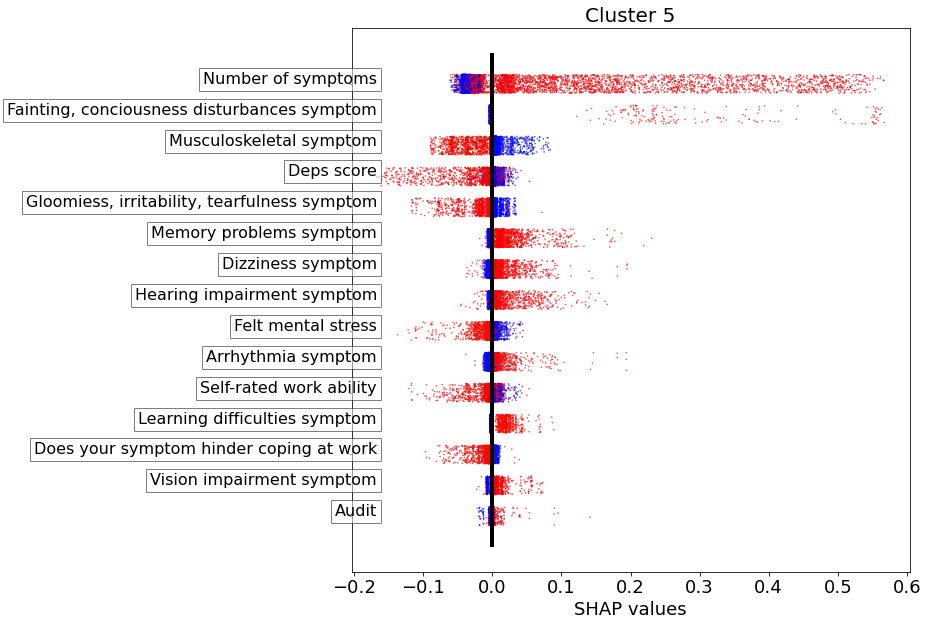


Figure S10. Shapley values (SHAP) for the 15 most important variables predicting cluster membership of Cluster 5 (Dizziness and sensory symptoms). Shapley values were calculated from the supervised classification model (XGBoost) that was trained for discriminating cluster 5 from all other clusters. The red points indicate higher employee-specific variable values than the average value of the variable and the blue points are lower employee-specific variable values than the average value of the variable. A longer distance between red and blue points indicates a higher capacity of a variable to change predicted cluster membership. The points at the right side of the black vertical line increase the probability of cluster membership. The points at the left side decrease the probability.


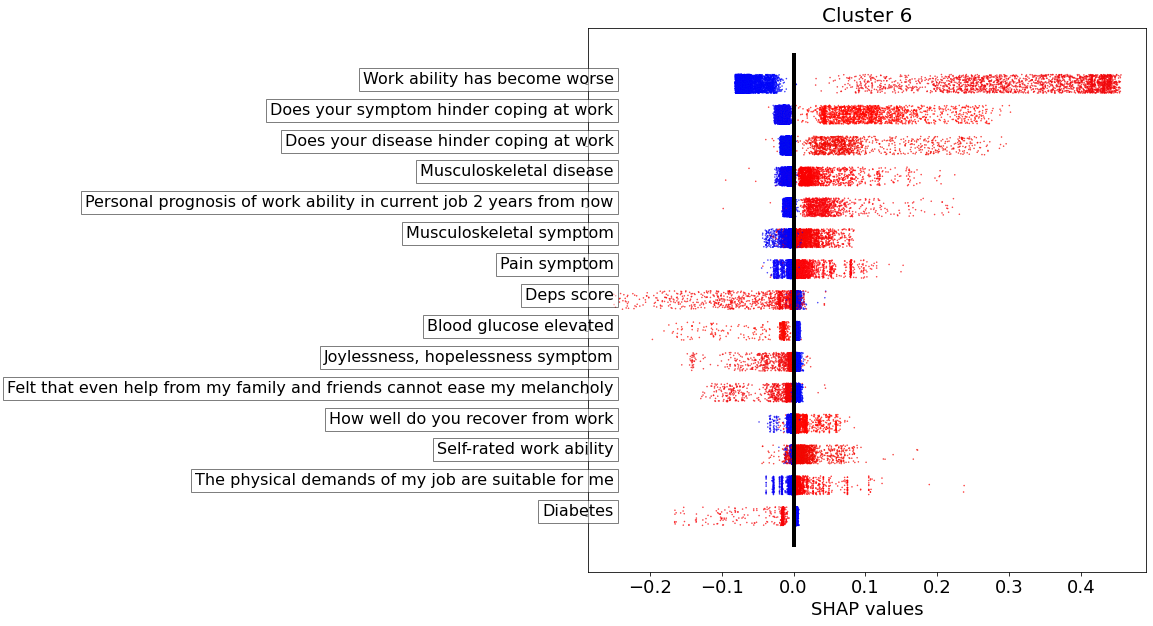


Figure S11. Shapley values (SHAP) for the 15 most important variables predicting cluster membership of Cluster 6 (Work ability). Shapley values were calculated from the supervised classification model (XGBoost) that was trained for discriminating cluster 6 from all other clusters. The red points indicate higher employee-specific variable values than the average value of the variable and the blue points are lower employee-specific variable values than the average value of the variable. A longer distance between red and blue points indicates a higher capacity of a variable to change predicted cluster membership. The points at the right side of the black vertical line increase the probability of cluster membership. The points at the left side decrease the probability.


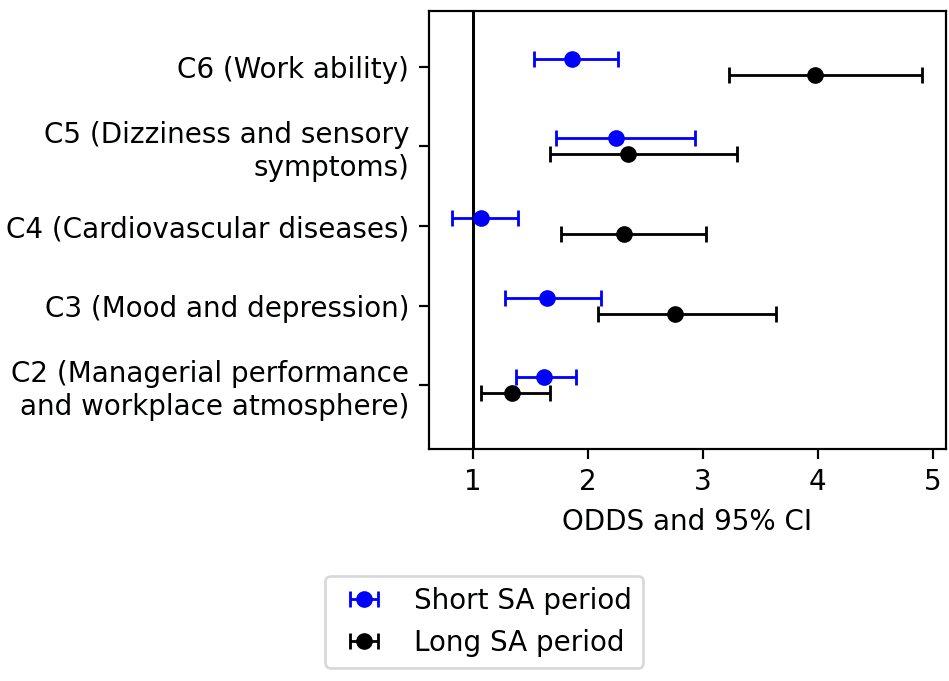


## Figure S12. Associations of clusters with repetitive short and long sickness absences in a Finnish occupational health cohort in 2011–2019.

Note. Odds ratio (OR) values and 95 % confidence intervals of the logistic regression models of the outcomes five short (1–10 days) sickness absence episodes during two years follow-up time or one or more long (>30 days) sickness absence episode during two years follow-up time. The reference cluster was the healthy cluster (Cluster 1).

## Figure S13. The distribution of industries among employees using Finla Occupational Health services in 2019 and the Finnish working population in 2022.

Official Statistics of Finland (OSF): Structural business and financial statement statistics [online publication]. ISSN=2342-6233.
